# Strategies to counteract adverse remodeling of vascular graft: A 3D view of current graft innovations

**DOI:** 10.3389/fbioe.2022.1097334

**Published:** 2023-01-10

**Authors:** Wei Tan, Parnaz Boodagh, Prakash Parthiban Selvakumar, Sean Keyser

**Affiliations:** ^1^ Department of Mechanical Engineering, University of Colorado Boulder, Boulder, CO, United States; ^2^ McGowan Institute for Regenerative Medicine, University of Pittsburgh, Pittsburgh, PA, United States; ^3^ Department of Mechanical Engineering, North Dakota State University, Fargo, ND, United States

**Keywords:** vascular grafts, adverse remodeling, impairment factor, scaffold technology, cell technology

## Abstract

Vascular grafts are widely used for vascular surgeries, to bypass a diseased artery or function as a vascular access for hemodialysis. Bioengineered or tissue-engineered vascular grafts have long been envisioned to take the place of bioinert synthetic grafts and even vein grafts under certain clinical circumstances. However, host responses to a graft device induce adverse remodeling, to varied degrees depending on the graft property and host’s developmental and health conditions. This in turn leads to invention or failure. Herein, we have mapped out the relationship between the design constraints and outcomes for vascular grafts, by analyzing impairment factors involved in the adverse graft remodeling. Strategies to tackle these impairment factors and counteract adverse healing are then summarized by outlining the research landscape of graft innovations in three dimensions—cell technology, scaffold technology and graft translation. Such a comprehensive view of cell and scaffold technological innovations in the translational context may benefit the future advancements in vascular grafts. From this perspective, we conclude the review with recommendations for future design endeavors.

## 1 Introduction

Vascular grafts are widely used for vascular surgeries. Their uses occur in a variety of forms, including vascular access for hemodialysis, as well as surgical bypass for diseased coronary artery, cerebral artery, or peripheral artery in the abdomen (e.g., aorto-iliac bypass) or in the leg (e.g., femoro-popliteal bypass), routing blood flow around an area of blockage or narrowing. A vascular graft can be a native artery or vein, or fabricated from synthetic or natural biomaterials. The current gold standard for a graft is autologous blood vessels such as a saphenous vein, which has better clinical outcomes than biomaterials-based grafts (Caliskan et al., 2020), even those made with tissue engineering approaches (Zilla et al., 2020). A vein fistula for dialysis access, for example, shows better primary patency than a polytetrafluoroethylene (PTFE) graft (Stegmayr et al., 2021). Similarly, for femoro-popliteal bypass, the 5-year patency rate of a vein graft is higher than a PTFE graft (van der Slegt et al., 2014). However, a quality vein suitable for grafting may be unavailable for many patients, particularly elders (Conte, 2012; AbuRahma, 2018). Also, to become a dialysis access, a vein must undergo a prolonged process to gain sufficient strength until its maturation into artery-like property. The maturation of a vein fistula requires several weeks up to months, during which compromised dialysis access may deteriorate patients’ health (Robbin et al., 2016). To make it worse, the incidence of fistula non-maturation varies between 20 and 60% (Kosa et al., 2015; Siddiqui et al., 2017).

Thus, bioengineered or tissue-engineered vascular grafts (TEVGs) have long been envisioned to take the place of synthetic grafts and even vein grafts under certain clinical circumstances. Selective TEVGs have gradually progressed into clinical trials, for both adult patients (Lawson et al., 2016; Kirkton et al., 2019) and pediatric patients with congenital heart diseases (Hibino et al., 2010; Drews et al., 2017; Sugiura et al., 2018; Drews et al., 2020), showing some promises despite yet limited successes. The clinical potentials of TEVGs, acellular or cellular, still require tremendous efforts to unveil (Zilla et al., 2020; Fang et al., 2021a). One possible route is to cater the TEVG development to a specific grafting need or a patient cohort, in which factors that impair healing or regeneration around the vascular graft can be well defined and targeted for graft design and engineering.

**FIGURE 1:**
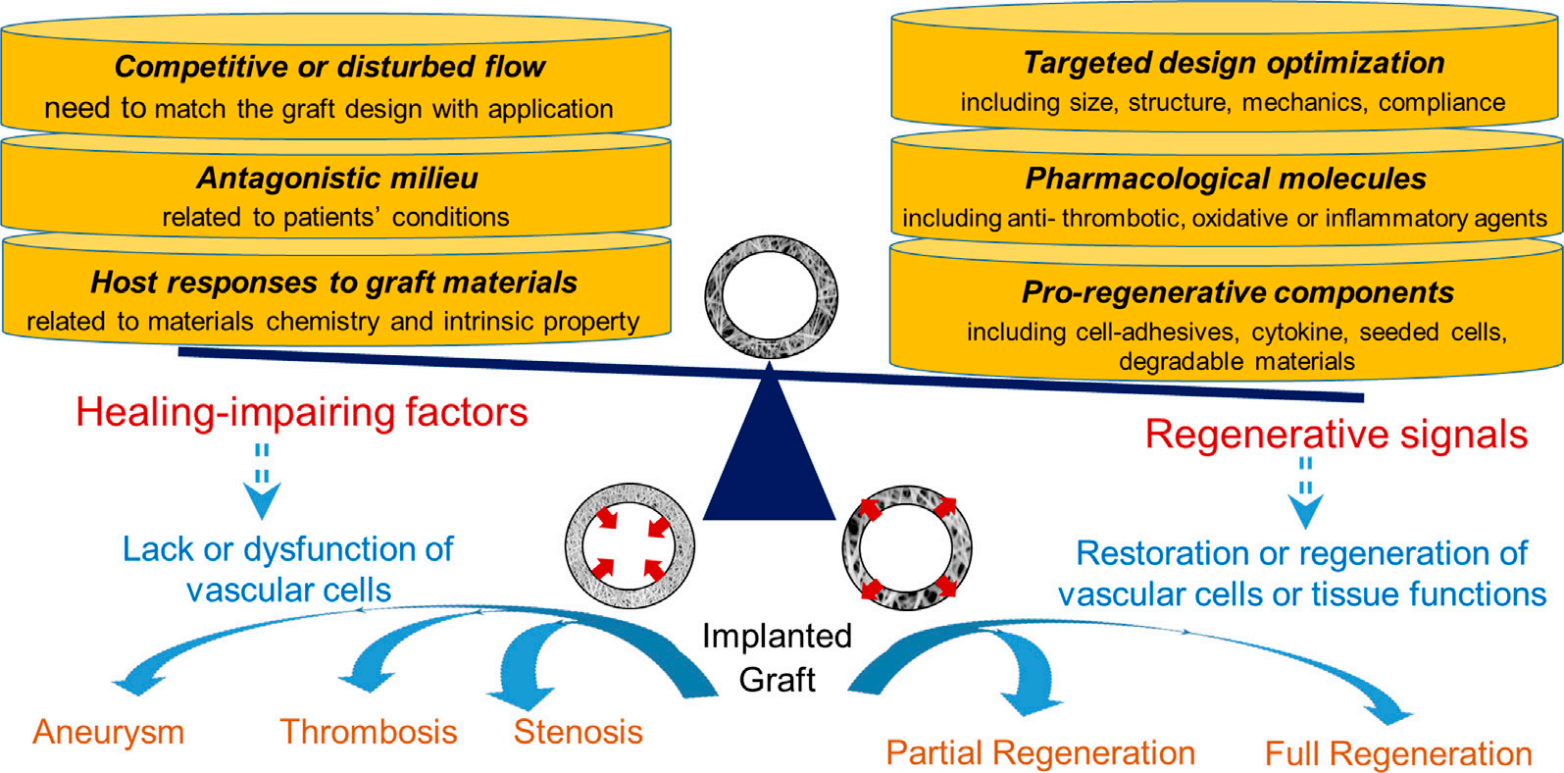
Illusration of impairment factors vs. regenerative signals for vascular grafts.

To this end, this review starts with analyzing healing- or regeneration-impairment factors, which are tackled by recent graft innovations. To withstand or override the influences of impairment factors, pro-healing and/or pro-regenerative environments could be precisely defined and built into a graft through structural optimizations, signal molecule incorporations, and integration of bioactive materials, cells or cell products. As illustrated in Figure 1, major healing-impairing factors are identified according to their roles in causing the dysfunction of a graft and/or neighboring blood vessels. A number of studies have been designed to reveal cause-effect relationships and unravel underlying molecular mechanisms. To counteract these impairing factors, a variety of strategies that promote design optimization, graft healing or regeneration have emerged. We focused a literature review on those published in recent years and placed them in the landscape of graft innovations in translation, scaffold and cell technologies. Essential to the future graft development is a continual effort to balance the multi-dimensional graft innovations with the fundamental mechanistic understandings underlying adverse graft remodeling.

## 2 Factors impairing healing or regeneration around vascular grafts

In clinic, graft outcomes in the treatment of vascular diseases are often determined by the size and location of the devices as well as the patients’ developmental and health conditions, besides the graft type (i.e., native vessel vs. synthetic graft). Herein, we examined these graft design constraints and associated clinical outcomes, by analyzing relevant impairment factors such as flow-graft interactions, cellular and molecular environments around grafts, and graft bioinertness.

### 2.1 Graft location, size and compliance: Related to flow-graft interactions

Abnormal blood flow in the graft has long been known as a key impairment factor that alters graft healing and overall patency (Schwarz et al., 2021). Computational flow dynamics tools, with the inputs of graft location, diameter, length and/or compliance together with graft design, have been applied to predict not only flow conditions but also consequent remodeling of a graft (Lu et al., 2014; Ramachandra et al., 2017; Donadoni et al., 2020). Variations in these physical characteristics of a graft delicately influence local fluid stresses in a blood vessel or a graft. Abnormal hemodynamic stresses may further result in impaired graft endothelialization, impaired graft healing and endothelial disruption in neighboring vessels (Chistiakov et al., 2017). Irregularities in the wall shear stress are known to induce endothelial dysfunctions, leading to negative clinical outcomes such as intimal hyperplasia, wherein smooth muscle cells (SMCs) grow to restrict the lumen of a vessel or a graft (Chistiakov et al., 2017; Ng et al., 2017; Totorean et al., 2019). Interestingly, in the case of dialysis access graft, hyperplasia develops predominantly in the vein or anastomotic regions, particularly toe and heel of distal anastomosis, where flow disturbances and altered hemodynamic factors are most prevalent (Sottiurai et al., 1989; Bassiouny et al., 1992). Quantitatively, low shear stress values (<.5 Pa) or oscillatory values (±.1–.2 Pa), as opposed to physiological values (1–2 Pa), were shown to induce endothelial dysfunction and subsequent hyperplasia development (Dhawan et al., 2010). The graft location determines flow environments around a graft (Chiu and Chien, 2011); abnormal flow patterns might occur in arteries with branches or curvatures or grafts such as looped vascular access or brachial arterial grafts as compared to aortic grafts.

The graft diameter is key to reach a hemodynamic balance. Bypass grafts with unfavorable diameter ratios to the original vessel can result in competitive flow, ultimately leading to graft failure. Competitive flows occur when a partially stenotic blood vessel is bypassed with a graft, but still retains some of its throughput, thereby competing with the newly grafted path and causing irregular flow through it (Gaudino et al., 2016). A high competitive flow decreases the amount of blood that reaches the graft, creating low shear stresses that increase the levels of hyperplasia at the graft’s anastomosis (Jin and Liu, 2019). Clinical findings suggest that more stenotic vessels with lower competitive flow, compared to less stenotic vessels, were better tolerated by coronary bypasses, and arterial grafts were more susceptible to competitive flow-induced impairment than venous grafts (Nordgaard et al., 2010; Gaudino et al., 2016; Sabik Joseph, 2016). The presence of chronic competitive flow is inversely related to internal mammary graft patency (Binns et al., 1989; Sabik et al., 2003). Intriguingly, saphenous vein graft patency did not decrease with increasing competitive flow (Ding et al., 2012; Swillens et al., 2012; Sugiura et al., 2018). Such difference could be attributed to cell and tissue differences between venous and arterial grafts (Harskamp et al., 2016; Sabik Joseph, 2016). Thus, the graft diameter in comparison with the original vessel is an important consideration for reducing flow-induced impairment, in particular for some graft types.

The roles of graft compliance and length in determining the graft patency are also well known (Skovrind et al., 2019). Compliance mismatch was recognized as a major contributor to graft failure (Tiwari et al., 2003). Though the influence of graft length on patency rates was insignificant for a similar grafting application (Tinica et al., 2018; Bosiers et al., 2020), applications that require long grafts, such as femoral artery lesion and hemodialysis access, often yielded low patency rates (Connors et al., 2011; Boutrous et al., 2019). This may be attributed to a wide range of biomechanical forces across a long graft with a large surface area. These forces consist of a complex loading combination from varying amounts of radial deformation, bending, extension/contraction, and torsion/flexion, which significantly impact the cell-graft interactions.

### 2.2 Patient’s conditions: Related to cellular and molecular environments around grafts

The graft failure in the cases of coronary bypass, dialysis access, or stent graft have been variably associated to age, diabetes mellitus, hypertension or other health conditions (Gaudino et al., 2017; AbuRahma, 2018; Misskey et al., 2018). Sex may serve as a possible compounding factor (Blum et al., 2021). Diabetes mellitus in particular causes a high risk of platelet aggregation in the blood and increases the release of von Willebrand factor, which promotes platelet aggregation and results in significant endothelial damage and arterial stiffening (Creager et al., 2003). This vascular intima damage coupled with the pathophysiological mechanism of diabetes leads to the increased formation of thrombosis (Reddy et al., 2013), and thus sooner obstruction of a graft such as arteriovenous fistula (Afsar and Elsurer, 2012). The primary failure of fistulas is associated with an enormous overall cost. Interestingly, arteriovenous fistula showed even higher primary failure rates than synthetic grafts in patients with diabetes mellites (Qin et al., 2016). To make it worse, failure of a fistula reduces the number of possible sites for an alternate access, exposing the patients to risks from salvaging attempts (Schinstock et al., 2011). Therefore, using hemodialysis access for diabetic patients as an example, it is clear that patients’ health conditions complicate cellular and molecular environments around grafts, worsening the ultimate fate of grafts, in particular biological grafts. For above-knee femoropopliteal bypass, diseases including diabetes and ischemia significantly reduced graft patency in both vein and PTFE grafts, but showing similar reduction rates for both types of grafts (Klinkert et al., 2004). Furthermore, during atherosclerosis such as coronary artery diseases, systemic biological factors (e.g., diabetes mellitus, dyslipidemia, sex, age, hypertension, and smoking) and local biological mechanisms (e.g., increased oxidative stress, vascular inflammation, and endothelial dysfunction) all contribute to the initial plaque formation and subsequent progression (Gaudino et al., 2017). These classic cardiovascular risk factors, particularly diabetes mellitus, seem to play a role in determining graft failure. For instance, the impact of diabetes mellitus on arterial and venous bypass grafts has been investigated by several groups, with most but not all, showing a detrimental effect on graft patency (Gaudino et al., 2017).

Besides comorbid diseases, the patency of a biological graft, compared to an inert synthetic graft, might be more influenced by cellular and molecular environments associated with older age. Older patient’s arteries tend to be more easily calcified making them more prone to abnormal shear stress, inflammation and other endothelial dysfunction (Tesauro et al., 2017). Due to significant comorbidities in elderly patients, a vein fistula is more likely to fail due to the deteriorating condition of blood vessels (Miller et al., 1999; Ponce, 2001; Lazarides et al., 2007). The use of synthetic PTFE grafts instead of vein fistula in elderly patients and those with compromised vessels might improve primary patency rates (Staramos et al., 2000; Rooijens et al., 2005). Clinical data point to advanced age as a significant factor in adversely affecting outcome of dialysis graft (Sugimoto et al., 2003; Moist et al., 2012). Meanwhile, almost half of all patients initiating dialysis are 65 years of age or older in the United States and the percentage of elderly patients is even higher in Canada and Europe (Ahmed and Catic, 2018), which makes the age as an important consideration for the graft design. It is increasingly recognized that an advanced age is associated with adverse graft remodeling and differentially altered remodeling of vein fistula and synthetic graft, likely through aged-related changes in vascular cells and molecular environments initiated by increases in pro-inflammatory cytokines, pro-calcification signaling, advanced glycation end products, and/or extracellular matrix stiffness. Similarly, when a bioengineered graft or TEVG include a cellular and/or molecular component, its design should consider adverse environments related to clinical conditions such as aging. In the case of vein fistula for dialysis access, its risk of failure is greatest among older patients and/or obese patients (Lok et al., 2006; Peterson et al., 2008; Shingarev et al., 2011).

### 2.3 Bioinertness of grafts

Graft bioinertness, or lack of biointegration, is another major impairment factor for long-term graft patency. It is mainly related to commercial grafts such as those made of PTFE or Dacron, but could be variedly applicable to different TEVGs, particularly prior to the completion of vascular tissue regeneration. Previous review articles have elaborated the main issues with the graft inertness, which include thrombosis, inflammation and intimal hyperplasia of a graft. Infection can also be a serious issue (Kirkton et al., 2018), in particular for dialysis graft requiring continuous needle access (Halbert et al., 2020; Kostokis and Loukopoulos, 2020; Stegmayr et al., 2021). A generally accepted root cause of these issues is the poor interaction of cells and the non-specific adsorption of proteins onto the graft, which lead to platelet aggregation but fail to form organized endothelium and/or matured smooth muscle. To circumvent these issues, bioengineering strategies including chemical coatings, materials modifications with peptides, proteins or other biomolecules, and seedings of endothelial cells or stem cells on inert or degradable grafts, have been utilized, with the goal of better integrating grafts with the native vasculature (Hielscher et al., 2018; Wang et al., 2020; Ham et al., 2021; Liu et al., 2021; Yu et al., 2021). Results with these strategies demonstrated improved endothelialization and/or inhibited thrombosis, inflammation, intimal hyperplasia, or infection. More recent approaches involve degradable, bioactive materials, as described below.

## 3 Counteracting adverse remodeling with regenerative signals

**FIGURE 2:**
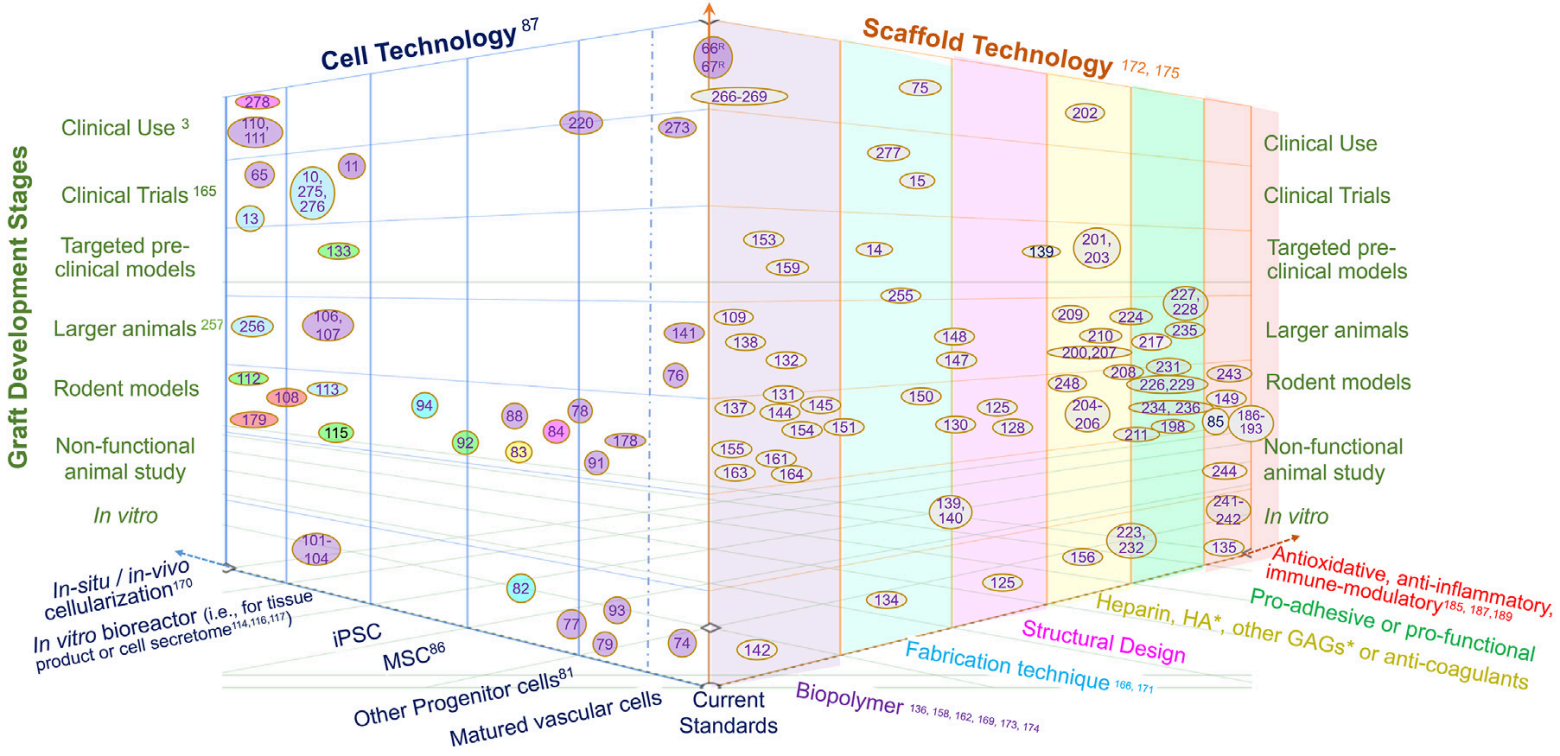
The landscape of vascular graft research in three dimensions, namely cell technology, scaffold technology and development stage. The citations of references used here are the numbered references listed in numerical order as they appear in the paper, as shown in Supplementary Material. The reference literatures are mapped in this landscape by locating research studies in the 3-dimensional space with reviews with various scopes labeled on the axes. The colors of oval boxes in the space of cell technology are correlated to the axis scaffold, showing the scaffold technology utilizied in these studies. Herein, “Current standards” refer to autologous graft (vein or artery) or bioinert graft (mostly PTFE-based); “Targeted preclinical models” refer to animal studies targeted at a clinical application such as dialysis access, Fontan graft, bypass for coronary, femoral or other arteries; “Larger animal” refer to animal models larger than rodents, such as rabbit, sheep, pig, dog and balloon; “Non-functional animal study” refer to non-vascular implants (often subcutaneous) or those used in *ex vivo* circulation; “*In situ* or *in vivo* cellularization” refer to the cellularization and/or tissue production by implanting acellular polymer; “Matured vascular cells” refer to vascular endothelial, smooth muscle and fibroblast cells; “Other progenitor cells” including endothelial progenitor cell and smooth muscle progenitor cell; “Biopolymer” includes biodegradable polymer , biologically-derived polymer, decellularized ECM or vessel, bioinert polymer, etc. *HA, hyaluronic acid; GAGs, glycosaminnoglycans.

To tackle above-mentioned factors involved in adverse graft remodeling, we summarize a three-thronged approach being taken in this field. A focused literature review of recent publications is taken to map out the research landscape of graft innovations in three dimensions, namely innovations in cell technology, scaffold technology and translation (Figure 2). Each referred study is a data point plotted on three axes in the attempt to show the relationship of the study in the context of the three innovations. Thus, a comprehensive perspective is taken for a combined view of both cell and scaffold technological innovations in the translational context.

### 3.1 Cell technology

The contribution of cell technology to counteract adverse remodeling is to enhance graft acceptance and integration with native vessels, including attenuating chronic inflammation, reducing thrombogenicity, and promoting fast healing and vascular regeneration. The cell technology includes the technology to isolate, culture and use different types of cells for vascular graft applications, the technology to recruit cells into grafts, and the technology to produce cell products crucial for regeneration. Herein, a brief overview is taken on each cell type used for vascular grafting, highlighting recent innovations in tissue bank, immune-tolerant cells, and bioreactors. Recent cell technology has been expanded to include the use of cell secretomes, while on the other end adding a range of *in situ* or *in vivo* cellularization technique into the realm of cell technology.

Autologous matured vascular cells, such as SMCs, endothelial cells and fibroblasts (McAllister et al., 2009; Syedain et al., 2011; Amensag and McFetridge, 2012), were employed for the TEVG creation in early preclinical and clinical studies. They are not immunogenic, but their disadvantages include invasive harvest of vascular cells, limited availability, varied quality, and low potential of replication and regeneration.

Progenitor cells, compared to matured cells, are isolated using less invasive procedures, from blood, bone marrow, umbilical cord, adipose or other tissues, and they demonstrate greater proliferative capacities despite their scarcity in tissues. For example, bone marrow-derived smooth muscle progenitor cells, compared to adult vascular SMCs, produced stronger and tougher TEVGs *in vitro*, and consequently progenitor seeded-grafts produced more organized elastin when implanted *in vivo* as jugular replacements in lambs (Swartz et al., 2005; Liu et al., 2007). Similarly, endothelial progenitor cells have been used to seed TEVG (Melchiorri et al., 2016; Muniswami et al., 2020; Tamma et al., 2020), but their high heterogeneity and rare presence could complicate the translation process (Munisso and Yamaoka, 2020). Mesenchymal stem cell (MSC), a type of progenitor cell with the potential of generating various vascular cell types (Gong and Niklason, 2008), lacks major histocompatibility complexes or other immune-stimulatory molecules while secreting anti-thrombotic and anti-inflammatory molecules, such as IL-10 (Fukunishi et al., 2018; Mirhaidari et al., 2020), and TGF-β receptor 1 (Lee et al., 2016). These characteristics make MSC an excellent allogeneic cell candidate for the TEVG production (Wang et al., 2016; Afra and Matin, 2020). In particular, adipose-derived MSCs are advantageous for their easy extraction in high quantities and high regenerative potency in a way unaffected by age (Harris et al., 2011; Zhang et al., 2011; Krawiec et al., 2016; Krawiec et al., 2017; Haskett et al., 2018). Finally, other new types of progenitor cells such as pericardial effusion-derived progenitor cells (Wang et al., 2021) can be potential candidates for cellularizing vascular grafts.

Induced pluripotent stem cells (iPSCs) have the potential of generating patient-specific SMCs, endothelial cells, and fibroblasts for TEVGs (Generali et al., 2019; Luo et al., 2020; Shi et al., 2021). The availability of iPSC-derived cell sources may eliminate the problems with variation in the cell quality and address the timing issue for “off-the-shelf” availability, both of which are concerns relevant to progenitor cells. Nevertheless, a bottleneck of using human iPSC derivatives is their tumorigenicity and immunogenicity (de Almeida et al., 2013). Thus, human leukocyte antigen-matched iPSC tissue banks would be a valuable source for personalized grafts (Jin et al., 2016; Petrus-Reurer et al., 2021). Additionally, genetically engineered master human iPSC lines, which give rise to immune-tolerant (immune-evasive or immune-suppressive) iPSC derivatives (Riolobos et al., 2013), might yield universal “off-the-shelf” cell sources for hypo-immunogenic TEVGs.

Cell seeding *in vitro* often requires an incubation time ranging from days to months in a dish or in a sophisticated bioreactor. Bioreactor designs vary from a rotary bioreactor for stem cell differentiation (Xing et al., 2017; Li et al., 2019a), a closed apparatus for standardizing cell technology (Kurobe et al., 2015), a biaxial stretching system for tissue matrix maturation (Huang et al., 2015; Huang et al., 2016), a semi-automated cell seeding device (Cunnane et al., 2020a), to a perfusion bioreactor for applying flow stresses (Melchiorri et al., 2016) and individualized graft conditioning (Håkansson et al., 2021). Another recent innovation in the *in vitro* cell seeding technology is to exploit 3D bioprinting techniques for printing patterned cell-laden hydrogels, utilizing a mixture of cells and hydrogel as the bioink to create vascular grafts with precisely-defined structures.

Recently, *in situ* or *in vivo* cellularization, utilizing human (or animal) body as a bioreactor, added a novel option into the cell technology for vascular grafts. This approach starts with placing a tube composed of Teflon (Wang et al., 2019a; Qiu et al., 2021), silicone (Fujita et al., 2020) or collagen (Nakayama et al., 2020), as a subcutaneous or peritoneal cavity implant or in an *ex vivo* location, allowing fibrous tissues to grow and form an tubular structure, which is then explanted and subsequently implanted as an autologous graft (Chen et al., 2019; Nakayama et al., 2020) or as an allograft after decellularization (Wang et al., 2019a; Fujita et al., 2020; Qiu et al., 2021). Preclinical or clinical studies showed some encouraging outcomes.

Last but not least is that the future cell technology for vascular grafts will surely see an increased use of stem cell secretomes (Chen et al., 2018; Cunnane et al., 2018; Cunnane et al., 2020b), as their therapeutic and regenerative values are being unveiled (Ahangar et al., 2020; Baruah and Wary, 2020; Nikfarjam et al., 2020; Tang et al., 2021) and recently exploited in other cardiovascular implants (Li et al., 2019b; Baruah and Wary, 2020; Hu et al., 2021).

### 3.2 Scaffold technology

The scaffold technology encompasses the techniques to improve the graft structure and mechanics and those to improve the biological integration. The former includes design innovations involving novel computational tools, biopolymers, or scaffold fabrication techniques ranging from decellularization to electrospinning and 3D printing. The scaffold technology for improved biological integration often involves the incorporation of a range of bioactive functional biomolecules on the surface or within a biopolymer, in order to modulate immune responses and/or to promote regeneration.

#### 3.2.1 Structural optimization and scaffold fabrication

**FIGURE 3:**
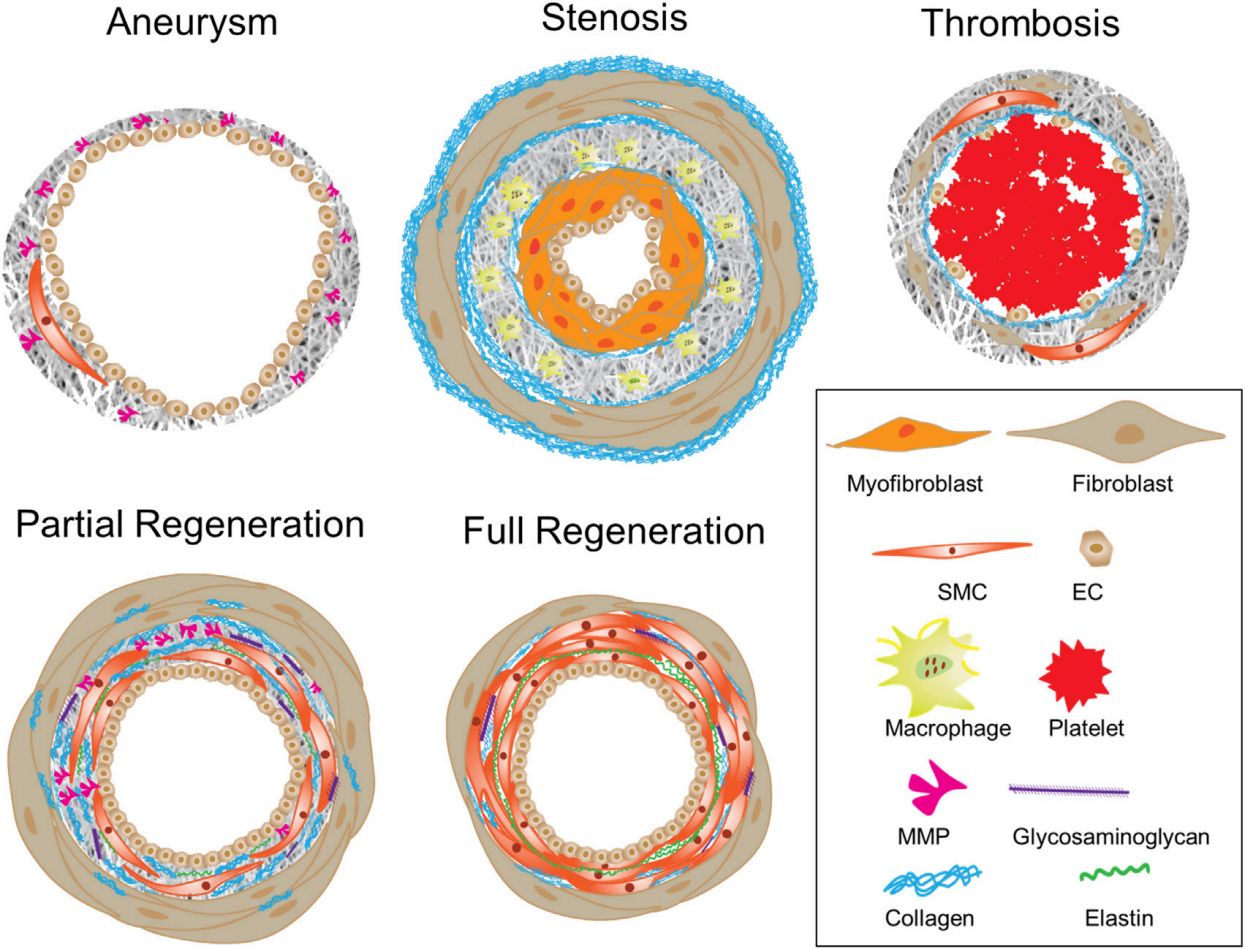
Adverse remodeling versus regeneration of a vascular graft.

As described in Section 2.1, the patency of vascular grafts is largely determined by the structural and mechanical interactions between blood flow and vascular graft, which continuously alter neotissue growth and graft remodeling. The neotissue growth and graft remodeling in turn change the flow-graft interactions. Therefore, computational models simulate such interactions as well as advanced statistical analyses of a parametric cause-effect relationship have been exploited to accelerate the rational optimization of vascular grafts (Keshavarzian et al., 2019; Tamimi et al., 2019; Khosravi et al., 2020). Recently, data-informed models have been developed to improve graft designs, even toward patient-specific design (Best et al., 2018), for desired graft performances in terms of lumen diameter, extracellular matrix (ECM) production, and levels of inflammation (Best et al., 2019; Drews et al., 2020). Model parameters comprise not only graft compliance, pore size, fiber diameter and degradation rate (Szafron et al., 2019; Tamimi et al., 2019; Furdella et al., 2021), but possibly also polymer chemistry, cytokine and other biological inputs (Szafron et al., 2018; Khosravi et al., 2020). Importantly, model outputs were compared to experimental outcomes and further predicting outcomes, which thus unravel mechano-biological mechanisms of neovessel formation and graft degradation *in vivo*. For example, models have been used to guide the design of mechanocompatible grafts (Furdella et al., 2021), to examine the role of inflammation activity using immuno-compromised animals (Szafron et al., 2018), or to parametrically explore graft narrowing (Drews et al., 2020; Khosravi et al., 2020). Besides computational models, statistical methods were used to reveal the roles of specialized design variables such as braiding angle, braiding density and coating structure in the graft development (Zbinden et al., 2020). Collectively, these studies have confirmed the well-accepted tissue engineering principle—the successful neovessel formation requires neotissue development to balance the scaffold degradation. However, such balance varies greatly with the animal model (Fukunishi et al., 2020). Lack of a balanced coordination over the time can result in adverse remodeling such as excessive degradation, impaired ECM synthesis, elevated inflammation and neotissue overgrowth in the lumen, leading to aneurysm (Best et al., 2019) or stenosis (Drews et al., 2020), and ultimately graft failure (Stowell et al., 2020). Figure 3 illustrates graft wall remodeling in various scenarios, where physiological routes ended up with partial or complete regeneration.

In parallel with the development of scaffold optimization tools, equally important is a rich availability of scaffold fabrication techniques and biopolymer choices, both of which promote parametric control for optimizations. Some recent developments in the graft fabrication include: 1) Shortening the production time for cell sheet self-assembly method (von Bornstädt et al., 2018); 2) loading drugs, anti-thrombogenic or pro-regenerative molecules for electrospun grafts or 3D printed grafts (Zhang et al., 2019; Domínguez-Robles et al., 2021); 3) refining decellularization protocols for reduced immunological responses (Schneider et al., 2018; Valencia-Rivero et al., 2019; Kimicata et al., 2020; Lopera Higuita et al., 2021); 4) improving the precision of pore generation in scaffold (Zhen et al., 2021); 5) enhancing recellularization for allogenic or xenogenic decellularized grafts (Dahan et al., 2017; Lin et al., 2019; Fayon et al., 2021); 6) expediting degradation with scaffold composition (Fukunishi et al., 2021) or textile technique (Fukunishi et al., 2019) to enhance matrix remodeling; 7) mimicking the structure and/or composition of vascular ECM using electrochemical fabrication (Nguyen et al., 2018) or an automated technology combining dip-spinning with solution blow spinning (Akentjew et al., 2019); 8) creating patient-specific grafts (Fukunishi et al., 2017); and 9) hybrid approaches, for example, combining electrospinning with decellularized matrices (Gong et al., 2016; Ran et al., 2019; Wu et al., 2019; Yang et al., 2019).

Biopolymers used for the graft construction kept diversifying the graft repertoire. For instance, the recent addition of shape memory polymer as a self-enclosable external support to a graft reduced stenosis (Yi et al., 2021). Currently, graft biopolymers consist of biologically-derived biomaterials such as silk fibroin (Zamani et al., 2017; Gupta et al., 2020; Tanaka et al., 2020), collagen (Li et al., 2017; Copes et al., 2019) and fibrin (Syedain et al., 2017), and synthetic polymers mostly degradable polymers such as polycaprolactone (Mrówczyński et al., 2014), polyglycolic acid (Sugiura et al., 2017), polyurethane (Zhen et al., 2021; Fathi-Karkan et al., 2022), polycarbonate urethane (Eilenberg et al., 2020), polylactic acid, and poly (l-lactide-co-ε-caprolactone) (Zhu et al., 2018). Hybrid graft materials composed of both biological polymers and synthetic polymers further leveraged the benefits offered by both materials for the mechanics and biological recognition of a graft. For further information, a number of reviews have provided historical views and in-depth analyses of graft fabrication techniques and graft polymers (Hiob et al., 2017; Pagel and Beck-Sickinger, 2017; Song et al., 2018; Chandra and Atala, 2019; Gentile et al., 2020; Niklason and Lawson, 2020; Syedain et al., 2020; Yuan et al., 2020; Gupta and Mandal, 2021; Heng et al., 2021; Zhang et al., 2021).

By and large, a long-term, dynamic, mechanistic view is taken into the design parameters and optimizations for vascular grafts, while continuous advancements in fabrication techniques and materials allow diverse choices and better controls over design parameters. A central finding in the graft optimization is that dynamically evolving mechanical properties can dictate the levels of inflammation and stress shielding of cells, and in turn affect mechanobiologically-mediated ECM production, remodeling and degradation (Szafron et al., 2019; Tamimi et al., 2019; Khosravi et al., 2020; Furdella et al., 2021). Therefore, adjusting the material chemistry and fabrication technique for desired dynamic profiles of graft mechanics in a specific clinical setting would be essential to future improvements.

#### 3.2.2 Starting right at the onset—molecular designs for anti-thrombotic and anti-inflammatory control

Events immediately occurring upon vascular grafting in the body involve endothelial injury platelet activation, and acute inflammation. Properly regulating these early events is crucial to the fate of a graft, either promoting regeneration or preventing failure from thrombosis or intimal hyperplasia (Roh et al., 2010; Tseng et al., 2014; Wissing et al., 2017). Talacua et al. (2015) showed that modulating the initial inflammatory phase in the first days remarkably improved the 3-month implantation outcome. In fact, the immune response is always pivotal to tissue regeneration: on one hand, it is indispensable for proper healing; on the other hand, extensive, prolonged inflammation prevent regeneration (Julier et al., 2017). Ideally, after acute inflammation, a vascular graft would start healing, gradually reach long-term structural stability, restore the normal vascular flow, and regenerate vascular functions. However, when a graft undergoes a healing process with a prolonged presence of inflammatory cells and non-vascular replacement tissues, fibrous formation and fibrous encapsulation may dominate, compromising graft mechanics, vascular flow and flow-dependent vascular cell functions and tissue regeneration (Boccafoschi et al., 2014).

Critical in the initial healing process is the macrophage activity, which determines ultimate graft remodeling or regeneration (Rodriguez-Soto et al., 2021). Macrophage functions are often performed through macrophage polarization (M1/M2) as well as their secreted factors including cytokines acting directly or indirectly on the vascular cells to regulate arteriogenesis (Boccafoschi et al., 2014). Cytokines such as IL-4 and IL-10 have been implicated in the transition of macrophages from a pro-inflammatory phenotype to an anti-inflammatory, pro-healing phenotype *in vivo* (Browne and Pandit, 2015). The biomaterials form, crosslinking level, degradability, hydrophilicity, topography, and materials choice of an implant can all affect the immune system, triggering varied inflammation (Boccafoschi et al., 2014; Browne and Pandit, 2015). Macrophage infiltration and polarization provides a strategy for predicting, detecting, and inhibiting stenosis in TEVGs (Szafron et al., 2018).

Because of the pivotal roles of macrophages at the onset of grafting (Zhang and King, 2022), recent strategies towards regulating macrophage functions include the use of immunomodulatory agents, drugs, cytokines, and cells such as progenitor cells. Immunomodulatory agents such as losartan (an angiotensin-II type1 receptor antagonist), zoledronate (a bisphosphonate), and aspirin-triggered resolvin D1, for example, significantly reduce macrophage infiltration or expedite inflammation resolution, thus attenuating graft stenosis and/or promoting regeneration (Ruiz-Rosado et al., 2018; Shi et al., 2019a; Chang et al., 2021). Grafts loaded with immunomodulatory cytokines, such as IL-4 (Tan et al., 2019) or human Wharton’s jelly matrix (rich in immunomodulatory cytokines) (Gupta et al., 2021), enhanced M2 macrophage percentage, which suppressed intimal hyperplasia (Tan et al., 2019) or promoted functional regeneration (Gupta et al., 2021). Similarly, immunomodulatory cells, either by *in vitro* cell seeding or by circulating cell recruitment *via* ligands immobilized to a graft, successfully regulated macrophage polarization and inflammation process. Fukunishi et al., for instance, seeded mononuclear cells onto electrospun TEVG scaffolds, which reduced platelet activation and attenuated graft stenosis through a higher M2 macrophage percentage and lower macrophage infiltration (Fukunishi et al., 2018). Lorentz et al. loaded microparticles with monocyte recruitment factor (C-C motif chemokine ligand 2) to induce tissue remodeling of vascular grafts (Lorentz et al., 2021). Shafiq et al. (2018) used Substance P and stromal cell–derived factor-1α peptide, both of which encouraged the recruitment of bone marrow cells to initiate healing and anti-inflammatory response. Similarly, Kim et al. (2019) also used Substance P for cell recruitment and accelerated regeneration.

Thrombosis is another early adverse event for a vascular graft and is caused by the lack of a functional endothelium which prevents the aggregation of blood components. Due to the essential roles of endothelial cells in the regulation of thrombogenesis, the *in vitro* endothelialization of a vascular graft was used prior to implantation. But for acellular grafts, if antithrombotic therapy is not used (Fang et al., 2021b), anti-fouling polymers or anticoagulants such as heparin must be attached to the graft surface for thrombogenic reduction through the prevention of platelet adhesion and/or activation (Radke et al., 2018). Long, flexible hydrophilic polymer chains can offer protein-repellent backgrounds for anti-coagulation (Thalla et al., 2012; Strang et al., 2014); multi-armed hydrophilic polymers further allow other functional molecules to be clickable to the graft surface (Iglesias-Echevarria et al., 2021). Binding of heparin to vascular grafts continues to be the most effective and widely-used method to prevent thrombosis, meanwhile heparin has anti-inflammatory effect and inhibitory effect on intima hyperplasia (Poterucha et al., 2017). Heparin binding can be *via* chemical conjugations or physical methods, but chemical bonding may compromise mechanical compatibility (Jiang et al., 2016). Regardless of graft materials or fabrication methods, heparinized grafts, including ePTFE grafts (Samson et al., 2016; Freeman et al., 2018), electrospun grafts (Qiu et al., 2017; Xu et al., 2018; Shi et al., 2019b; Matsuzaki et al., 2021; Zhu et al., 2021), and decelluarized grafts (Kong et al., 2019), all outperformed those without heparin in terms of graft patency, showing critical roles of heparin in the thromboresistance and *in situ* endothelialization. Besides heparin, anticoagulation methods include the incorporation of anti-thrombotic drugs (Lee et al., 2022), hyaluronic acid (Dimitrievska et al., 2020) or more sophisticated ECM secreted by thrombospondin-2 knockout cells on decellularized aorta grafts (Kristofik et al., 2017).

In summary, regulating the platelet activity and immune response to a graft is pivotal to initiate graft healing and determine regeneration—galvanize or depress it. The final stage of immune responses involves the coordination of macrophages, endothelial cells and SMCs. The migration and proliferation of the latter two result in the regeneration of new functional arterial tissues. Disruption to the collaborative efforts of these cells as well as any negative exogenous factors such as infection, stresses or environmental factors, can lead to thrombosis and hyperplasia, and eventually graft failure. Therefore, factors influencing graft inflammation at the onset may largely determine its long-term patency. Additional factors affecting the long-term graft patency include physiochemical properties of biomaterials, such as hydrophobicity, stiffness and bioinertness. The current graft material, PTFE, for example, in some cases, induces severe graft calcification, resulting in suboptimal mid-term and long-term outcomes.

#### 3.2.3 Biological functionalization of a graft for vascular regeneration

**FIGURE 4:**
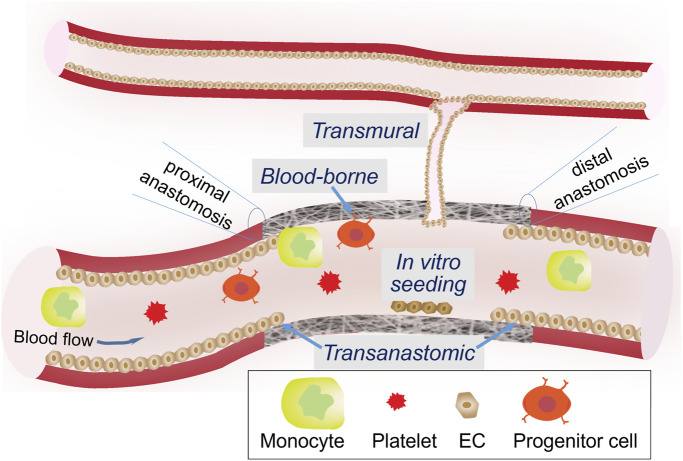
Possible mechanisms underlying graft endothelization.

Due to indispensable, protective roles of endothelial cells throughout the vasculature, it is widely accepted that the endothelialization of a vascular graft is of the utmost importance to graft designers. Reinstitution of a continuous endothelium monolayer on the lumen surface of a graft is the best way to avoid the activation of coagulation cascade and progression of intimal hyperplasia (Heath, 2017). Four endothelialization mechanisms have been identified (Figure 4). The pros and cons of each are summarized in Table 1. Transanastomotic growth refers to the migration of matured endothelial cells from the native vessel onto the luminal surface of the graft across the anastomoses, where a vascular graft is connected to a native vessel. It served as a major endothelialization mechanism in animal models, but did not extend beyond 1 cm in humans (Zilla et al., 2007; Zilla et al., 2020). Therefore, to endothelialize the middle section of a longer graft, other mechanisms must be considered. Transmural growth occurs through cellular and vascular infiltration from an adjacent blood vessel (Pennel et al., 2013). Blood-borne growth is through cells in the circulation with the capacity of differentiation into endothelial phenotypes, such as endothelial progenitor cells. Such endothelialization modality requires a strong binding between the circulating cell and the graft surface, which may be offered by progenitor-specific antibodies or ligand (Lu et al., 2013; Hao et al., 2020) or adhesive peptides (Choi et al., 2016; Hao et al., 2017; Liu et al., 2021) immobilized on the lumen surface. Since all three *in situ* mechanisms are still insufficient to create a continuous endothelium throughout a long vascular graft in human patients, *ex vivo* or *in vitro* seeding of matured vascular cells (Dahan et al., 2017), progenitor/stem cells (Olausson et al., 2012; Ardila et al., 2019), or genetically modified autologous cells expressing fibulin-5 and VEGF, onto the graft lumen have been employed (Sánchez et al., 2018).

**TABLE 1:** Summary of comparisons among four endothelialization mechanisms.

Endothelialization mechanisms	Pros	Cons
Trans-anastomotic growth	Effective in small animals; *in situ*	Irrelevant or ineffective in most clinical applications; complicated by inflammatory responses
Transmural growth	Essential for graft healing; *in situ*	Inconsistent results due to the complexity of angiogenic process; influenced by physical properties of the scaffold
Blood borne growth	Regenerative poten-tial; *in situ* seeding	Scarcity of circulating cells and limited regenerative capability of cells in the circulation
*in vitro* seeding	Formation of a confluent cell layer	No “off-the-shelf”; possible cell loss *in vivo*; Efficiency and outcome depend on cell source, culture and seeding techniques

**FIGURE 5:**
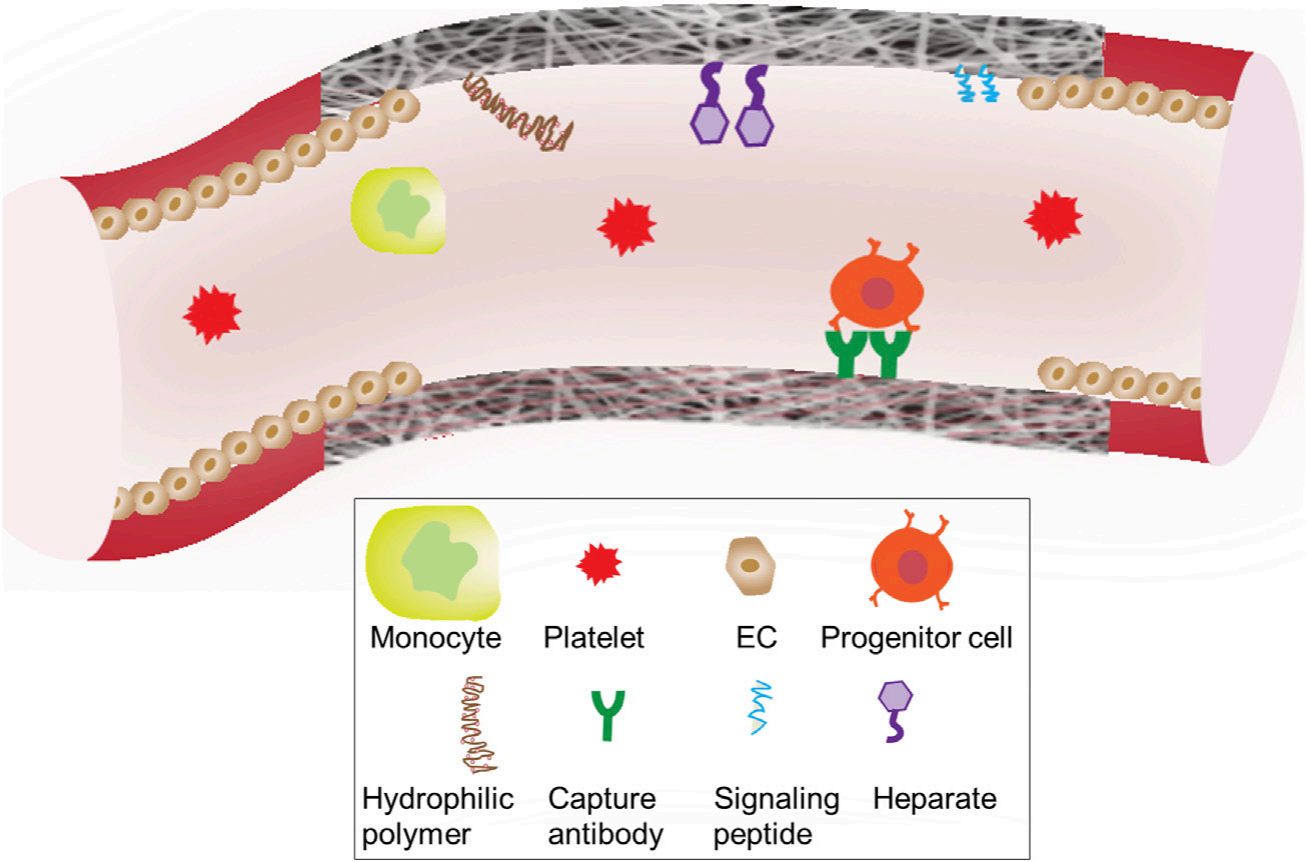
Surface engineering strategies of grafts Biomolecules conjugated to the surface graft materials are designed to interact with the blood components including blood cells, progenitor cells and platelets.

For *in situ* endothelialization and *in situ* smooth muscle regeneration, strategies to functionalize graft lumen or an inner layer, have been under continuous development to improve the regeneration speed and quality. To encourage the adhesion, migration, differentiation and proliferation of matured or progenitor-derived vascular cells, bioactive molecules utilized for graft functionalization include: (a) growth factors, (b) genes, (c) peptides, and (d) antioxidants. Figure 5 illustrates functionalization strategies for graft endothelialization.

One of the most adopted growth factors is VEGF (Koobatian et al., 2016; Wang et al., 2019b). Despite an early study showing undesired inflammation and intimal hyperplasia on a decellularized vascular graft with a coating of FGF and VEGF (Heidenhain et al., 2011), a number of recent studies using these or other angiogenic or stem cell-homing molecules demonstrated their positive influences on graft performances. For example, the incorporation of triple factors, VEGF, bFGF, and SDF-1α into a two-layered graft was found to improve endothelialization, SMC layer formation, and thus graft patency compared with VEGF alone (Antonova et al., 2021). Allogeneic decellularized vascular scaffold grafted with VEGF, bFGF, and heparin exhibited similar mechanical properties to the natural vessels, and positive expression of factor VIII and α2-actin (Kong et al., 2019). Smith et al. (2019) and Smith et al. (2020) developed a vascular graft based on small intestinal submucosa with immobilized heparin and VEGF on the graft lumen to capture VEGF receptor-expressing circulating angiogenic cells from the blood, which promoted the formation of artery-mimic structure with endothelial and medial layers. Besides VEGF, other signaling mediators, including brain-derived neurotrophic factors (Zeng et al., 2012), dickkopf-3 glycoprotein (Issa Bhaloo et al., 2018), and SDF-1α (Shafiq et al., 2018; Wang et al., 2019c), were used to improve graft patency through progenitor cell homing and/or paracrine mechanisms for graft endothelialization and smooth muscle formation. Furthermore, these cell-recruiting molecules were used in combination with anticoagulant and immunomodulatory molecules. For example, the PTFE graft covalently coupled with heparin, SDF-1α and CD47 reduced thrombogenicity, facilitated the recruitment of progenitor cells, and alleviated the immune responses (Gao et al., 2017). Indeed, fast, high-quality *in situ* vascular regeneration could benefit from an exquisite graft design orchestrating the functions of multiple signaling molecules.

The gene incorporation into a graft can also effectively enhance vascular regeneration. Genes such as miRNAs and vectors encoding VEGF receptor-2 ligands (Hytönen et al., 2019) were used instead of functional proteins or peptides. In particular, miRNAs, such as miRNA-126 which accelerates the synthesis of VEGF and FGF and miRNA-145 which modulates the contractile SMC phenotype, were loaded into grafts: miRNA-126 alone was found to accelerate graft endothelialization (Zhou et al., 2016), while dual miRNA loading further improved contractile SMC regeneration and promoted vascular ECM formation (Wen et al., 2020). However, the lack of cell-specific transfection and release kinetics as well as potential toxicity and inflammation are several challenges for the gene approach.

The antioxidant approach could address both regenerative need for a functional endothelium and therapeutic need for inhibiting neointimal growth or calcification, which is related to oxidative stress-caused cellular damage (Gaudino et al., 2017; Washington and Bashur, 2017). A natural antioxidant, oxide (NO) is consistently secreted by endothelial cells, providing essential signaling to prevent thrombosis, SMC proliferation or intimal hyperplasia (Napoli et al., 2006; Strijdom et al., 2009; Devine et al., 2020). Therefore, NO-producing mechanisms, including NO-releasing coating and NO-generating coating such as NO-catalytic bioactive coating that generates NO from endogenous S-nitrosothiols, are integrated into vascular grafts (Wang et al., 2015; Tang et al., 2018; Dou et al., 2020; Li et al., 2020; Enayati et al., 2021) and stents (Yang et al., 2015; Yang et al., 2020), to promote endothelialization and reduce restenosis. Another type of antioxidant used was poly (1,8-octamethylenecitrate-co-cysteine), which was found to inhibit the calcification of decellularized grafts (Jiang et al., 2017).

## 4 Translation of vascular grafts for clinics: Success measures

Essential to the future development is continual efforts to balance the mechanistic understandings underlying adverse graft remodeling in clinic with the innovations in grafting technologies. Translational innovations are as important as innovations in cell technology and scaffold technology. Assessment tests and preclinical models for vascular grafts are to be properly matched to the targeted clinical application (Zilla et al., 2020), in terms of the anatomic structure such as graft diameter, length and looping, graft functions such as regular needle access for dialysis grafts, and regeneration potential related to clinical use, patient’s age and disease condition. Ideally, grafts with regenerated tissues could also simulate vasoreactivity and pharmacological responses of a native vessel, for the long-term success (Strobel et al., 2018).

### 4.1 Device function tests

Related to tubular vascular implants, FDA guidance documents have specified testing criteria for a number of physical and mechanical properties of grafts, including porosity, burst strength, compliance, elastic modulus, water permeability, compliance, and kink diameter (ANSI, 2010). Vascular grafts for a specific application such as arteriovenous graft access may require additional guidelines for function testing (Vascular Access Society, 2021). For example, when the synthetic grafts are placed a loop configuration, e.g., for vascular access, preventing graft kinking is crucial (Wu et al., 2020). Nevertheless, all the test guidelines refer to traditional, permanent graft materials. When biodegradable materials, synthetic or natural, are used to construct grafts, it may be necessary to test the temporal changes of physical and mechanical properties, in order to avoid devastating events such as aneurysm. Besides physical and mechanical properties, *in vitro* device tests also include biological function assessment. As TEVG improvements focus on the graft development by attending to the main causes of graft failure—thrombus formation and intimal hyperplasia, *in vitro* thrombogenicity tests as well as proliferation and migration tests of vascular cells have been widely employed.

### 4.2 Preclinical assessment with animals

The goal of TEVG regeneration studies is to translate new findings to human clinical therapy, which relies on animal models to generate enormous data useful to the translation. Ideally, the selection of animal model considers the animal’s analog close to human patients in terms of similarities in anatomical, structural, pathological and/or functional aspects. Immunodeficient animal models further allow long-term accommodation of humanized vascular grafts (Itoh et al., 2019). To evaluate the biochemical, structural and functional similarities between a TEVG implant and a native artery in animals, measures taken on the implants include ultrasonic scan, flow Doppler and X-ray/angiography, as well as on explant analyses, such as mechanics (i.e., strength, compliance), histological analyses such as geometry (i.e., lumen size, thickness), layer structure, cells (e.g., inflammatory cells, circulating cells, vascular endothelial cells and SMC, cells related to fibrotic or calcified remodeling), ECM (e.g., elastin, collagen types I/III/IV), and biomolecules through gene and/or protein assays.

A closer analog to clinical conditions allows one to better predict graft healing and regeneration in clinic. A huge discrepancy exists between endothelialization outcomes in animal models and those occurring in clinic (Zilla et al., 2007; Sánchez et al., 2018; Cai et al., 2021). Despite increased graft assessments in different animal models, most studies still use young, healthy small animals. It could be attributed to drastic differences in the vascular anatomy and physiology between human and rodents, which are used for evaluations in the majority of animal studies. The re-endothelization of a vein graft was reported to complete after several weeks of post-surgery in rodents, but re-endothelization duration in human took much longer. More important than the late presence of an endothelium on TEVG in human is a mismatch in the endothelialization mechanism—mainly trans-anastomotic mechanism in short grafts for rodents versus mainly transmural or blood-borne endothelialization in much longer grafts for humans (Zilla et al., 2007). In the clinical context of PTFE grafts, trans-anastomotic endothelialization covers only the immediate peri-anastomotic graft region in patients. Based on the analysis in a senescent non-human primate model, Zilla et al. (2007) and Zilla et al. (2020), concluded that a major issue with the development of prosthetic vascular grafts lied in wrong animal models, which led to wrong questions and no healing. Large animal models such as sheep, pigs, and baboons, though less available and more costly, mimic human physiology, in terms of vascular anatomy, thrombogenicity, inflammatory responses and regenerative potentials (Fukunishi et al., 2016; Rothuizen et al., 2016; Ju et al., 2017; Ma et al., 2017; Syedain et al., 2017; Liu et al., 2018; Schleimer et al., 2018; Fang et al., 2021b).

Overall, the design of future preclinical studies would better address the clinical relevance, for example, dialysis access (Gage and Lawson, 2017; Ong et al., 2017) and meso-Rex bypass (Maxfield et al., 2017), by providing further insight into the mechanisms through experimental designs that are aligned with the purpose and scope of specific clinical applications. In selecting animal models that prematurely terminate adverse remodeling events, the factors that impair vascular regeneration around grafts in clinic may remain largely overlooked. Besides the type of animal models, the significant “impairment factors” as listed in Section 2 include age and disease conditions, e.g., diabetes and ischemia. Additionally, temporal and spatial characterizations of the host responses in animals would be crucial to improve our mechanistic understandings of TEVG remodeling.

### 4.3 Clinical trials and potential commercialization values

Clinical trials are a true success measure and a determinant to the commercial value of any graft innovation. Prior trials have been performed on three types of grafts: Biological grafts (i.e., xenografts or allografts), bioreactor-manufactured grafts, and readily implantable grafts. Though trial results are encouraging and enlightening for future TEVG strategies, the overall success is still limited.

Biological grafts, referring to acellular blood vessels from human or animal (e.g., bovine) origin, have undergone clinical trials for decades. Bovine carotid artery grafts, such as those commercialized as Artegraft™, were used as arteriovenous fistula in patients undergoing chronic hemodialysis. These grafts outperformed PTFE grafts, though incrementally (Hutchin et al., 1975; Kennealey et al., 2011; Arhuidese et al., 2017). Lindsey et al. (2018) did a 15-year follow-up study on Artegaft used in lower extremity bypass surgery of 120 patients, yielding positive results for patency and salvage limb rates. Recently, the concern regarding the immunologic sensitization of artery xenografts in patients was also addressed (Dyer-Kindy et al., 2020). Similar to an artery graft, a vein xenograft such as bovine vein-derived ProCol™ demonstrated their candidacy as an alternative to synthetic grafts through clinical trials in both hemodialysis patients and patients with critical limb ischemia for 14–20 months (Hatzibaloglou et al., 2004; Schmidli et al., 2004). Vein allografts derived from human cadaveric veins were also a possible alternative. Cryopreserved allografts were used in 90 patients, showing similar patency, more resistant to infection but significantly more susceptible to aneurysms, when compared to PTFE grafts (Madden et al., 2004). More recently, 15 vein allografts seeded with autologous endothelial cells were used in coronary artery bypass surgery with 12 patients, showing patency up to 32 months (Herrmann et al., 2019). Autologous stem cells were also used to recellularize vein allografts and successfully tried on one pediatric patient for vascular vein shunts without the need for immunosuppression (Olausson et al., 2012). Overall, decades of clinical trials have shown that an artery- or vein-based xenografts or allografts can be considered as an alternative to synthetic PTFE or Dacron grafts. However, significant improvements over these graft products, in particular decellularization and recellularization as well as deproteinization and surface engineering to address complications- and/or rejections-derived from immune responses (Merola et al., 2017), are needed before they may gain a bit market share.

Bioreactor-manufactured grafts produce TEVG by culturing human autologous cells and/or allogeneic cells with or without a scaffold in a bioreactor with controlled physical, mechanical and/or biological environments. Clinical trials have illuminated promises and challenges with current methods. Lawson et al. (2016) seeded allogeneic human VSMCs onto polyglycolic acid scaffold, subjected the cell-loaded scaffold to pulsatile cyclic distension for 8 weeks before decellularization for final acellular implant. The patency of these grafts (Humacyte™) on 60 hemodialysis patients was 63% at 6 months and 28% at 18 months, with no evidence of immune response or aneurysm formation. From this trial, 16 tissue samples were acquired from 16 to 200 weeks for the characterization of vascular remodeling, showing recellularization of grafts by non-inflammatory host progenitor and vascular cells (Kirkton et al., 2019). A recent follow-up report provided 5-year (phase II) results on 11 patients who completed the study (Jakimowicz et al., 2022). Results showed that one patient maintained primary patency, and 10 maintained secondary patency. Like PTFE grafts, such acellular grafts showed better clinical outcomes when used as above-knee, femoral-to-popliteal arterial bypass conduits in 20 patients (Gutowski et al., 2020). As an example for scaffold-free cell-engineered graft, Cytograft™ employed patients’ fibroblasts to create an autologous TEVG through a prolonged fabrication procedure, either finishing with autologous endothelial seeding or implanting as it is. Its clinical trial with hemodialysis grafts on ten patients showed 1-month patency of 78% and 6-month patency of 60% (McAllister et al., 2009). Later, L’Heureux’s group modified the graft production into an “off-the-shelf” design—allogeneic Lifeline™ graft. Prior to implantation in patients for hemodialysis access, frozen cell-sheet based scaffolds were thawed, rehydrated and seeded with autologous endothelial cells the luminal side. All three implanted grafts worked well till 11 months (Wystrychowski et al., 2014).

More readily implantable grafts are desirable in clinic, as they require minimal or no need of cell culture time, relying on *in vivo* degradation, remodeling, regeneration and maturation. One recent approach is embedding a biotube in subcutaneous spaces of a patient for months, using patient’s body to fabricate a graft. This has been used successfully as a pulmonary artery substitute in pediatric patients (Kato et al., 2016; Fujita et al., 2020; Nakatsuji et al., 2021; Higashita et al., 2022). Another approach was employing biodegradable scaffolds in combination with autologous bone marrow mononuclear cells. Their clinical trials investigated these TEVGs in congenital heart surgery (Sugiura et al., 2018). Researchers observed postoperative growth of a TEVG as well as frequent graft narrowing in pediatric patients, but there was no graft-related mortality during the 11-year follow-up period.

## 5 Challenge and future

**FIGURE 6:**
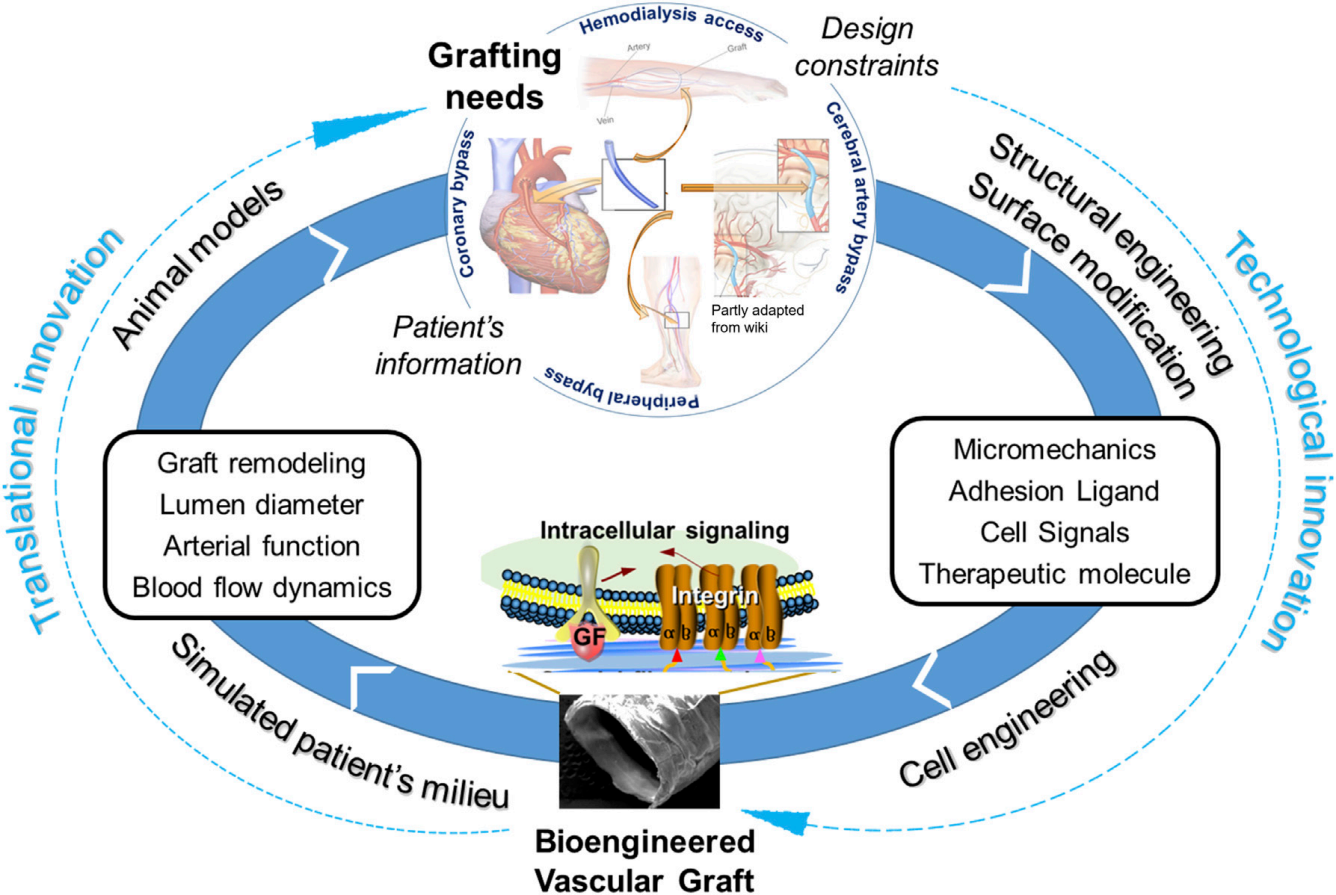
Coupling of technological innovations with translational innovations to tackle adverse remodeling of vascular grafts for clinical applications.

In conclusion, we reviewed the studies in the context of three innovations, i.e., cell technology, scaffold technology, and translational stages. Regenerative medicine has demonstrated great promises in the TEVG context. Future endeavors require the technological innovations to be coupled with translational innovations, in order to tackle the adverse remodeling of vascular grafts (Figure 6). This is critical to our fundamental understandings of the regenerative mechanisms and eventual transfer of the understandings into improved products for clinical practices. The complex nature of vascular remodeling and regeneration mechanisms in the spatial and temporal dimensions demands more sophisticated designs of *in vitro* studies and animal models. A combined approach employing both experimental and computational tools could significantly expedite the determination of optimal design parameters in a disease- or host-specific condition. From a perspective of clinical translation and graft commercialization, the considerations about costs and graft processing logistics become more and more important and are also highlighted in other recent reviews and studies. To address scientific questions to the point of clinical relevance, it is critical to highlight and increase the use of appropriate cell models, such as 3D cell models in diseased conditions, appropriate animal models, such as senescent animals, diseased animals, animals with slow vascular regenerative rates. Additionally, to more quantitatively and systematically understand the mechanisms, it would be helpful to standardize protocols such as sampling times across studies to obtain critical mechanobiological modeling inputs such as material degradation, inflammation, growth/remodeling/regeneration parameters.

Also, it is expected that the adaptation and integration of bioengineered or tissue-engineered vascular grafts to human patients are heterogeneous because of cells’ varied ability to adapt to new microenvironments. A comparable case of graft heterogeneity in clinic is the use of vein grafts. Some veins are poor remodelers due to lack of adaptation, which had a 13-fold increased risk of failure at 2 years compared with “robust remodelers” (Owens et al., 2015). One factor in this lack of adaptation is biological incompatibility between cells and host endothelium. Cellular heterogeneity arises due to reasons not fully understood yet. For example, iPSC-derived arterial endothelial cells exhibit functional differences from iPSC-derived venous endothelial cells, including nitric oxide production and elongation under shear stress (Rosa et al., 2019). At present, the limitation of cellular heterogeneity is also a direct consequence of difficulty in identifying and precisely isolating progenitor cells which has limited their use in TEVGs. Therefore, improving methods of cell isolation and differentiation as well as reducing heterogeneity between cells in grafts and host vessels could be a solution to better integrate TEVGs into native vasculature.

We acknowledge several limitations of this review study, which include the lack of in-depth analyses and justifications of graft design, fabrication, biomaterials, and molecular mechanisms underlying graft failure and integration.

## Abbreviations

ECM, extracellular matrix; iPSCs, induced pluripotent stem cells; MSC, mesenchymal stem cell; NO, nitric oxide; PTFE, polytetrafluoroethylene; SMC, smooth muscle cell; TEVG, tissue-engineered vascular grafts.
