# Supplementary material for: Strategies to counteract adverse remodeling of vascular graft: A 3D view of current graft innovations
Source: Front Bioeng Biotechnol. 2023 Jan 10;10:1097334. doi: 10.3389/fbioe.2022.1097334 (PMC9871289; doi:10.3389/fbioe.2022.1097334)
Supplement: Supplementary file 1 [file Table1.pdf]

Supplementary material:

The citations of references used in Figure 2 are the numbered references listed in numerical order as they appear in the paper, as below:

1. Caliskan E, de Souza DR, Böning A, et al. Saphenous vein grafts in contemporary coronary artery bypass graft surgery. *Nat Rev Cardiol*. 2020;17(3):155-169. doi:10.1038/s41569-019-0249-3
2. Zilla P, Deutsch M, Bezuidenhout D, Davies NH, Pennel T. Progressive Reinvention or Destination Lost? Half a Century of Cardiovascular Tissue Engineering. *Front Cardiovasc Med*. 2020;7:159. doi:10.3389/fcvm.2020.00159
3. Stegmayr B, Willems C, Groth T, et al. Arteriovenous access in hemodialysis: A multidisciplinary perspective for future solutions. *Int J Artif Organs*. 2021;44(1):3-16. doi:10.1177/0391398820922231
4. van der Slegt J, Steunenberg SL, Donker JMW, et al. The current position of precuffed expanded polytetrafluoroethylene bypass grafts in peripheral vascular surgery. *J Vasc Surg*. 2014;60(1):120-128. doi:10.1016/j.jvs.2014.01.062
5. Conte MS. Diabetic Revascularization: Endovascular Versus Open Bypass—Do We Have the Answer? *Semin Vasc Surg*. 2012;25(2):108-114. doi:10.1053/j.semvascsurg.2012.04.004
6. AbuRahma AF. When Are Endovascular and Open Bypass Treatments Preferred for Femoropopliteal Occlusive Disease? *Ann Vasc Dis*. 2018;11(1):25-40. doi:10.3400/avd.ra.18-00001
7. Robbin ML, Greene T, Cheung AK, et al. Arteriovenous Fistula Development in the First 6 Weeks after Creation. *Radiology*. 2016;279(2):620-629. doi:10.1148/radiol.2015150385
8. Kosa SD, Al-Jaishi AA, Moist L, Lok CE. Preoperative vascular access evaluation for haemodialysis patients. *Cochrane Database Syst Rev*. 2015;(9):CD007013. doi:10.1002/14651858.CD007013.pub2
9. Siddiqui MA, Ashraff S, Carline T. Maturation of arteriovenous fistula: Analysis of key factors. *Kidney Res Clin Pract*. 2017;36(4):318-328. doi:10.23876/j.krcp.2017.36.4.318
10. Lawson JH, Glickman MH, Ilzecki M, et al. Bioengineered human acellular vessels for dialysis access in patients with end-stage renal disease: two phase 2 single-arm trials. *Lancet Lond Engl*. 2016;387(10032):2026-2034. doi:10.1016/S0140-6736(16)00557-2
11. Kirkton RD, Santiago-Maysonet M, Lawson JH, et al. Bioengineered human acellular vessels recellularize and evolve into living blood vessels after human implantation. *Sci Transl Med*. 2019;11(485). doi:10.1126/scitranslmed.aau6934
12. Drews JD, Miyachi H, Shinoka T. Tissue-engineered vascular grafts for congenital cardiac disease: Clinical experience and current status. *Trends Cardiovasc Med*. 2017;27(8):521-531. doi:10.1016/j.tcm.2017.06.013
13. Sugiura T, Matsumura G, Miyamoto S, Miyachi H, Breuer CK, Shinoka T. Tissue-engineered Vascular Grafts in Children With Congenital Heart Disease: Intermediate Term Follow-up. *Semin Thorac Cardiovasc Surg*. 2018;30(2):175-179. doi:10.1053/j.semtcvs.2018.02.002
14. Drews JD, Pepper VK, Best CA, et al. Spontaneous reversal of stenosis in tissue-engineered vascular grafts. *Sci Transl Med*. 2020;12(537). doi:10.1126/scitranslmed.aax6919

15. Hibino N, McGillicuddy E, Matsumura G, et al. Late-term results of tissue-engineered vascular grafts in humans. *J Thorac Cardiovasc Surg*. 2010;139(2):431-436, 436.e1-2. doi:10.1016/j.jtcvs.2009.09.057
16. Fang S, Ellman DG, Andersen DC. Review: Tissue Engineering of Small-Diameter Vascular Grafts and Their In Vivo Evaluation in Large Animals and Humans. *Cells*. 2021;10(3):713. doi:10.3390/cells10030713
17. Schwarz EL, Kelly JM, Blum KM, et al. Hemodynamic performance of tissue-engineered vascular grafts in Fontan patients. *NPJ Regen Med*. 2021;6(1):38. doi:10.1038/s41536-021-00148-w
18. Donadoni F, Pichardo-Almarza C, Homer-Vanniasinkam S, Dardik A, Díaz-Zuccarini V. Multiscale, patient-specific computational fluid dynamics models predict formation of neointimal hyperplasia in saphenous vein grafts. *J Vasc Surg Cases Innov Tech*. 2020;6(2):292-306. doi:10.1016/j.jvscit.2019.09.009
19. Lu DY, Chen EY, Wong DJ, et al. Vein graft adaptation and fistula maturation in the arterial environment. *J Surg Res*. 2014;188(1):162-173. doi:10.1016/j.jss.2014.01.042
20. Ramachandra AB, Humphrey JD, Marsden AL. Gradual loading ameliorates maladaptation in computational simulations of vein graft growth and remodelling. *J R Soc Interface*. 2017;14(130):20160995. doi:10.1098/rsif.2016.0995
21. Chistiakov DA, Orekhov AN, Bobryshev YV. Effects of shear stress on endothelial cells: go with the flow. *Acta Physiol*. 2017;219(2):382-408. doi:10.1111/apha.12725
22. Ng Jaryl, Bourantas Christos V., Torii Ryo, et al. Local Hemodynamic Forces After Stenting. *Arterioscler Thromb Vasc Biol*. 2017;37(12):2231-2242. doi:10.1161/ATVBAHA.117.309728
23. Totorean AF, Hudrea IC. Local Hemodynamics in Coronary Bypass in the Presence of Competitive Flow and Different Diameter Ratios Between Graft and Host Artery. In: Lhotska L, Sukupova L, Lacković I, Ibbott GS, eds. *World Congress on Medical Physics and Biomedical Engineering 2018*. IFMBE Proceedings. Springer; 2019:767-771. doi:10.1007/978-981-10-9035-6_141
24. Bassiouny HS, White S, Glagov S, Choi E, Giddens DP, Zarins CK. Anastomotic intimal hyperplasia: Mechanical injury or flow induced. *J Vasc Surg*. 1992;15(4):708-717. doi:10.1016/0741-5214(92)90019-5
25. Sottiurai VS, Yao JST, Batson RC, Sue SL, Jones R, Nakamura YA. Distal Anastomotic Intimal Hyperplasia: Histopathologic Character and Biogenesis. *Ann Vasc Surg*. 1989;3(1):26-33. doi:10.1016/S0890-5096(06)62381-9
26. Dhawan SS, Nanjundappa RPA, Branch JR, et al. Shear stress and plaque development. *Expert Rev Cardiovasc Ther*. 2010;8(4):545-556. doi:10.1586/erc.10.28
27. Chiu JJ, Chien S. Effects of Disturbed Flow on Vascular Endothelium: Pathophysiological Basis and Clinical Perspectives. *Physiol Rev*. 2011;91(1):10.1152/physrev.00047.2009. doi:10.1152/physrev.00047.2009
28. Gaudino M, Niccoli G, Scalone G, et al. Competitive Flow and Coronary Artery Bypass Grafts. In: Țintoiu IC, Underwood MJ, Cook SP, Kitabata H, Abbas A, eds. *Coronary Graft Failure: State of the Art*. Springer International Publishing; 2016:277-284. doi:10.1007/978-3-319-26515-5_25
29. Jin C, Liu Y. Influence of Competitive Flow Caused by Different Stenosis on Coronary Artery Bypass Hemodynamics and PIV Study. *Mol Cell Biomech*. 2019;16(S1). Accessed May 8, 2020. http://tspsubmission.com/index.php/mcb/article/view/5728

30. Nordgaard H, Swillens A, Nordhaug D, et al. Impact of competitive flow on wall shear stress in coronary surgery: computational fluid dynamics of a LIMA–LAD model. *Cardiovasc Res*. 2010;88(3):512-519. doi:10.1093/cvr/cvq210
31. Sabik Joseph F. Should Coronary Artery Bypass Grafting Be Performed in Patients With Moderate Stenosis of the Left Anterior Descending Coronary Artery? *Circulation*. 2016;133(2):111-113. doi:10.1161/CIRCULATIONAHA.115.020084
32. Binns RL, Ku DN, Stewart MT, Ansley JP, Coyle KA. Optimal graft diameter: Effect of wall shear stress on vascular healing. *J Vasc Surg*. 1989;10(3):326-337. doi:10.1016/0741-5214(89)90449-7
33. Sabik JF, Lytle BW, Blackstone EH, Khan M, Houghtaling PL, Cosgrove DM. Does competitive flow reduce internal thoracic artery graft patency? *Ann Thorac Surg*. 2003;76(5):1490-1497. doi:10.1016/S0003-4975(03)01022-1
34. Ding J, Liu Y, Wang F, Bai F. Impact of Competitive Flow on Hemodynamics in Coronary Surgery: Numerical Study of ITA-LAD Model. Computational and Mathematical Methods in Medicine. doi:https://doi.org/10.1155/2012/356187
35. Swillens A, De Witte M, Nordgaard H, et al. Effect of the degree of LAD stenosis on “competitive flow” and flow field characteristics in LIMA-to-LAD bypass surgery. *Med Biol Eng Comput*. 2012;50(8):839-849. doi:10.1007/s11517-012-0927-3
36. Harskamp RE, Alexander JH, Ferguson TB, et al. Frequency and Predictors of Internal Mammary Artery Graft Failure and Subsequent Clinical Outcomes: Insights From the Project of Ex-vivo Vein Graft Engineering via Transfection (PREVENT) IV Trial. *Circulation*. 2016;133(2):131-138. doi:10.1161/CIRCULATIONAHA.115.015549
37. Sabik Joseph F. Should Coronary Artery Bypass Grafting Be Performed in Patients With Moderate Stenosis of the Left Anterior Descending Coronary Artery? *Circulation*. 2016;133(2):111-113. doi:10.1161/CIRCULATIONAHA.115.020084
38. Skovrind I, Harvald EB, Juul Belling H, Jørgensen CD, Lindholt JS, Andersen DC. Concise Review: Patency of Small-Diameter Tissue-Engineered Vascular Grafts: A Meta-Analysis of Preclinical Trials. *Stem Cells Transl Med*. 2019;8(7):671-680. doi:10.1002/sctm.18-0287
39. Tiwari A, Cheng KS, Salacinski H, Hamilton G, Seifalian AM. Improving the patency of vascular bypass grafts: The role of suture materials and surgical techniques on reducing anastomotic compliance mismatch. *Eur J Vasc Endovasc Surg*. 2003;25(4):287-295. doi:10.1053/ejvs.2002.1810
40. Tinica G, Chistol RO, Enache M, Constantin MML, Ciocoiu M, Furnica C. Long-term graft patency after coronary artery bypass grafting: Effects of morphological and pathophysiological factors. *Anatol J Cardiol*. 2018;20(5):275-282. doi:10.14744/AnatolJCardiol.2018.51447
41. Bosiers MJ, Panuccio G, Bisdas T, et al. Longer bridging stent-grafts in iliac branch endografting does not worsen outcome and expands its applicability, even in concomitant diseased hypogastric arteries. *J Cardiovasc Surg (Torino)*. 2020;61(2). doi:10.23736/S0021-9509.18.10504-0
42. Connors G, Todoran TM, Engelson BA, Sobieszczyk PS, Eisenhauer AC, Kinlay S. Percutaneous revascularization of long femoral artery lesions for claudication: patency over 2.5 years and impact of systematic surveillance. *Catheter Cardiovasc Interv Off J Soc Card Angiogr Interv*. 2011;77(7):1055-1062. doi:10.1002/ccd.22802

43. Boutrous ML, Alvarez AC, Okoye OT, Laws JC, Jacobs DL, Smeds MR. Stent-Graft Length Is Associated with Decreased Patency in Treatment of Central Venous Stenosis in Hemodialysis Patients. *Ann Vasc Surg*. 2019;59:225-230. doi:10.1016/j.avsg.2019.01.024
44. Gaudino M, Antoniades C, Benedetto U, et al. Mechanisms, Consequences, and Prevention of Coronary Graft Failure. *Circulation*. 2017;136(18):1749-1764. doi:10.1161/CIRCULATIONAHA.117.027597
45. Misskey J, Faulds J, Sidhu R, Baxter K, Gagnon J, Hsiang Y. An age-based comparison of fistula location, patency, and maturation for elderly renal failure patients. *J Vasc Surg*. 2018;67(5):1491-1500. doi:10.1016/j.jvs.2017.08.080
46. Blum KM, Roby LC, Zbinden JC, et al. Sex and Tamoxifen confound murine experimental studies in cardiovascular tissue engineering. *Sci Rep*. 2021;11(1):8037. doi:10.1038/s41598-021-87006-3
47. Creager Mark A., Lüscher Thomas F., null null, Cosentino Francesco, Beckman Joshua A. Diabetes and Vascular Disease. *Circulation*. 2003;108(12):1527-1532. doi:10.1161/01.CIR.0000091257.27563.32
48. Reddy MA, Tak Park J, Natarajan R. Epigenetic Modifications in the Pathogenesis of Diabetic Nephropathy. *Semin Nephrol*. 2013;33(4):341-353. doi:10.1016/j.semnephrol.2013.05.006
49. Afsar B, Elsurer R. The primary arteriovenous fistula failure—a comparison between diabetic and non-diabetic patients: glycemic control matters. *Int Urol Nephrol*. 2012;44(2):575-581. doi:10.1007/s11255-011-9978-x
50. QIN HY, JIA P, LIU H. Nursing Strategies for Patients with Chronic Renal Failure Undergoing Maintenance Hemodialysis Treatment by Arteriovenous Fistula. *Iran J Public Health*. 2016;45(10):1270-1275.
51. Schinstock CA, Albright RC, Williams AW, et al. Outcomes of Arteriovenous Fistula Creation after the Fistula First Initiative. *Clin J Am Soc Nephrol*. 2011;6(8):1996-2002. doi:10.2215/CJN.11251210
52. Klinkert P, Post PN, Breslau PJ, van Bockel JH. Saphenous Vein Versus PTFE for Above-Knee Femoropopliteal Bypass. A Review of the Literature. *Eur J Vasc Endovasc Surg*. 2004;27(4):357-362. doi:10.1016/j.ejvs.2003.12.027
53. Tesauro M, Mauriello A, Rovella V, et al. Arterial ageing: from endothelial dysfunction to vascular calcification. *J Intern Med*. 2017;281(5):471-482. doi:10.1111/joim.12605
54. Ponce P. Vascular access for dialysis in the elderly. *Int Urol Nephrol*. 2001;33(3):571-573. doi:10.1023/A:1019550307514
55. Miller PE, Tolwani A, Luscy CP, et al. Predictors of adequacy of arteriovenous fistulas in hemodialysis patients. *Kidney Int*. 1999;56(1):275-280. doi:10.1046/j.1523-1755.1999.00515.x
56. Lazarides MK, Georgiadis GS, Antoniou GA, Staramos DN. A meta-analysis of dialysis access outcome in elderly patients. *J Vasc Surg*. 2007;45(2):420-426.e2. doi:10.1016/j.jvs.2006.10.035
57. Staramos DN, Lazarides MK, Tzilalis VD, Ekonomou CS, Simopoulos CE, Dayantas JN. Patency of autologous and prosthetic arteriovenous fistulas in elderly patients. *Eur J Surg*. 2000;166(10):777-781. doi:10.1080/110241500447407
58. Rooijens PPGM, Burgmans JPJ, Yo TI, et al. Autogenous radial-cephalic or prosthetic brachial-antecubital forearm loop AVF in patients with compromised vessels? A randomized, multicenter study of the patency of primary hemodialysis access. *J Vasc Surg*. 2005;42(3):481-487. doi:10.1016/j.jvs.2005.05.025

59. Sugimoto K, Higashino T, Kuwata Y, Imanaka K, Hirota S, Sugimura K. Percutaneous transluminal angioplasty of malfunctioning Brescia-Cimino arteriovenous fistula: analysis of factors adversely affecting long-term patency. *Eur Radiol*. 2003;13(7):1615-1619. doi:10.1007/s00330-002-1764-9
60. Moist LM, Lok CE, Vachharajani TJ, et al. Optimal Vascular Access in the Elderly Patient. *Semin Dial*. 2012;25(6):640-648. doi:10.1111/sdi.12037
61. Ahmed FA, Catic AG. Decision-Making in Geriatric Patients with End-Stage Renal Disease: Thinking Beyond Nephrology. *J Clin Med*. 2018;8(1):5. doi:10.3390/jcm8010005
62. Lok CE, Allon M, Moist L, Oliver MJ, Shah H, Zimmerman D. Risk Equation Determining Unsuccessful Cannulation Events and Failure to Maturation in Arteriovenous Fistulas (REDUCE FTM I). *J Am Soc Nephrol*. 2006;17(11):3204-3212. doi:10.1681/ASN.2006030190
63. Peterson WJ, Barker J, Allon M. Disparities in Fistula Maturation Persist Despite Preoperative Vascular Mapping. *Clin J Am Soc Nephrol*. 2008;3(2):437-441. doi:10.2215/CJN.03480807
64. Shingarev R, Maya ID, Barker-Finkel J, Allon M. Arteriovenous Graft Placement in Predialysis Patients: A Potential Catheter-Sparing Strategy. *Am J Kidney Dis*. 2011;58(2):243-247. doi:10.1053/j.ajkd.2011.01.026
65. Kirkton RD, Prichard HL, Santiago-Maysonet M, Niklason LE, Lawson JH, Dahl SLM. Susceptibility of ePTFE vascular grafts and bioengineered human acellular vessels to infection. *J Surg Res*. 2018;221:143-151. doi:10.1016/j.jss.2017.08.035
66. Halbert RJ, Nicholson G, Nordyke RJ, Pilgrim A, Niklason L. Patency of ePTFE Arteriovenous Graft Placements in Hemodialysis Patients: Systematic Literature Review and Meta-Analysis. *Kidney360*. 2020;1(12):1437-1446. doi:10.34067/KID.0003502020
67. Kostokis ID, Loukopoulos I. Comparison Between Bovine Carotid Artery Graft and Polytetrafluoroethylene Graft for Haemodialysis Vascular Access: A Systemic Review and Meta-Analysis. *J Vasc Surg*. 2020;72(5):1814. doi:10.1016/j.jvs.2020.08.012
68. Ham HO, Haller CA, Su G, et al. A rechargeable anti-thrombotic coating for blood-contacting devices. *Biomaterials*. 2021;276:121011. doi:10.1016/j.biomaterials.2021.121011
69. Liu Y, Mahara A, Kambe Y, Hsu YI, Yamaoka T. Endothelial cell adhesion and blood response to hemocompatible peptide 1 (HCP-1), REDV, and RGD peptide sequences with free N-terminal amino groups immobilized on a biomedical expanded polytetrafluorethylene surface. *Biomater Sci*. 2021;9(3):1034-1043. doi:10.1039/d0bm01396j
70. Wang D, Xu Y, Wang L, et al. Expanded Poly(tetrafluoroethylene) Blood Vessel Grafts with Embedded Reactive Oxygen Species (ROS)-Responsive Antithrombogenic Drug for Elimination of Thrombosis. *ACS Appl Mater Interfaces*. 2020;12(26):29844-29853. doi:10.1021/acsami.0c07868
71. Yu L, Newton ER, Gillis DC, et al. Coating small-diameter ePTFE vascular grafts with tunable poly(diol-co-citrate-co-ascorbate) elastomers to reduce neointimal hyperplasia. *Biomater Sci*. 2021;9(15):5160-5174. doi:10.1039/d1bm00101a
72. Hielscher D, Kaebisch C, Braun BJV, Gray K, Tobiasch E. Stem Cell Sources and Graft Material for Vascular Tissue Engineering. *Stem Cell Rev Rep*. 2018;14(5):642-667. doi:10.1007/s12015-018-9825-x
73. Amensag S, McFetridge PS. Rolling the Human Amnion to Engineer Laminated Vascular Tissues. *Tissue Eng Part C Methods*. 2012;18(11):903-912. doi:10.1089/ten.tec.2012.0119

74. Syedain ZH, Meier LA, Bjork JW, Lee A, Tranquillo RT. Implantable arterial grafts from human fibroblasts and fibrin using a multi-graft pulsed flow-stretch bioreactor with noninvasive strength monitoring. *Biomaterials*. 2011;32(3):714-722. doi:10.1016/j.biomaterials.2010.09.019
75. McAllister TN, Maruszewski M, Garrido SA, et al. Effectiveness of haemodialysis access with an autologous tissue-engineered vascular graft: a multicentre cohort study. *Lancet Lond Engl*. 2009;373(9673):1440-1446. doi:10.1016/S0140-6736(09)60248-8
76. Swartz DD, Russell JA, Andreadis ST. Engineering of fibrin-based functional and implantable small-diameter blood vessels. *Am J Physiol Heart Circ Physiol*. 2005;288(3):H1451-1460. doi:10.1152/ajpheart.00479.2004
77. Liu JY, Swartz DD, Peng HF, Gugino SF, Russell JA, Andreadis ST. Functional tissue-engineered blood vessels from bone marrow progenitor cells. *Cardiovasc Res*. 2007;75(3):618-628. doi:10.1016/j.cardiores.2007.04.018
78. Muniswami DM, Reddy LVK, Amirtham SM, et al. Endothelial progenitor/stem cells in engineered vessels for vascular transplantation. *J Mater Sci Mater Med*. 2020;31(12):119. doi:10.1007/s10856-020-06458-7
79. Melchiorri AJ, Bracaglia LG, Kimerer LK, Hibino N, Fisher JP. In Vitro Endothelialization of Biodegradable Vascular Grafts Via Endothelial Progenitor Cell Seeding and Maturation in a Tubular Perfusion System Bioreactor. *Tissue Eng Part C Methods*. 2016;22(7):663-670. doi:10.1089/ten.TEC.2015.0562
80. Tamma R, Ruggieri S, Annese T, Ribatti D. Vascular Wall as Source of Stem Cells Able to Differentiate into Endothelial Cells. In: Turksen K, ed. *Cell Biology and Translational Medicine, Volume 7: Stem Cells and Therapy: Emerging Approaches*. Advances in Experimental Medicine and Biology. Springer International Publishing; 2020:29-36. doi:10.1007/5584_2019_421
81. Munisso MC, Yamaoka T. Circulating endothelial progenitor cells in small-diameter artificial blood vessel. *J Artif Organs*. 2020;23(1):6-13. doi:10.1007/s10047-019-01114-6
82. Gong Z, Niklason LE. Small-diameter human vessel wall engineered from bone marrow-derived mesenchymal stem cells (hMSCs). *FASEB J*. 2008;22(6):1635-1648. doi:10.1096/fj.07-087924
83. Mirhaidari GJM, Barker JC, Zbinden JC, et al. Tissue Engineered Vascular Graft Recipient Interleukin 10 Status Is Critical for Preventing Thrombosis. *Adv Healthc Mater*. 2020;9(24):e2001094. doi:10.1002/adhm.202001094
84. Fukunishi T, Best CA, Ong CS, et al. Role of Bone Marrow Mononuclear Cell Seeding for Nanofiber Vascular Grafts. *Tissue Eng Part A*. 2018;24(1-2):135-144. doi:10.1089/ten.TEA.2017.0044
85. Lee YU, de Dios Ruiz-Rosado J, Mahler N, et al. TGF-β receptor 1 inhibition prevents stenosis of tissue-engineered vascular grafts by reducing host mononuclear phagocyte activation. *FASEB J Off Publ Fed Am Soc Exp Biol*. 2016;30(7):2627-2636. doi:10.1096/fj.201500179R
86. Afra S, Matin MM. Potential of mesenchymal stem cells for bioengineered blood vessels in comparison with other eligible cell sources. *Cell Tissue Res*. 2020;380(1):1-13. doi:10.1007/s00441-019-03161-0
87. Wang L, Hu J, Sorek CE, Chen EY, Ma PX, Yang B. Fabrication of tissue-engineered vascular grafts with stem cells and stem cell-derived vascular cells. *Expert Opin Biol Ther*. 2016;16(3):317-330. doi:10.1517/14712598.2016.1118460

88. Haskett DG, Saleh KS, Lorentz KL, et al. An exploratory study on the preparation and evaluation of a "same-day" adipose stem cell-based tissue-engineered vascular graft. *J Thorac Cardiovasc Surg*. 2018;156(5):1814-1822.e3. doi:10.1016/j.jtcvs.2018.05.120
89. Zhang P, Moudgill N, Hager E, et al. Endothelial differentiation of adipose-derived stem cells from elderly patients with cardiovascular disease. *Stem Cells Dev*. 2011;20(6):977-988. doi:10.1089/scd.2010.0152
90. Harris LJ, Abdollahi H, Zhang P, McIlhenny S, Tulenko TN, DiMuzio PJ. Differentiation of adult stem cells into smooth muscle for vascular tissue engineering. *J Surg Res*. 2011;168(2):306-314. doi:10.1016/j.jss.2009.08.001
91. Krawiec JT, Liao HT, Kwan LL, et al. Evaluation of the stromal vascular fraction of adipose tissue as the basis for a stem cell-based tissue-engineered vascular graft. *J Vasc Surg*. 2017;66(3):883-890.e1. doi:10.1016/j.jvs.2016.09.034
92. Krawiec JT, Weinbaum JS, Liao HT, et al. In Vivo Functional Evaluation of Tissue-Engineered Vascular Grafts Fabricated Using Human Adipose-Derived Stem Cells from High Cardiovascular Risk Populations. *Tissue Eng Part A*. 2016;22(9-10):765-775. doi:10.1089/ten.TEA.2015.0379
93. Wang JN, Kan CD, Lin SH, Chang KC, Tsao S, Wong TW. Potential of Autologous Progenitor Cells and Decellularized Porcine Artery Matrix in Construction of Tissue-engineered Vascular Grafts. *Organogenesis*. Published online August 18, 2021:1-13. doi:10.1080/15476278.2021.1963603
94. Generali M, Casanova EA, Kehl D, et al. Autologous endothelialized small-caliber vascular grafts engineered from blood-derived induced pluripotent stem cells. *Acta Biomater*. 2019;97:333-343. doi:10.1016/j.actbio.2019.07.032
95. Shi X, He L, Zhang SM, Luo J. Human iPS Cell-derived Tissue Engineered Vascular Graft: Recent Advances and Future Directions. *Stem Cell Rev Rep*. 2021;17(3):862-877. doi:10.1007/s12015-020-10091-w
96. Luo J, Qin L, Zhao L, et al. Tissue-Engineered Vascular Grafts with Advanced Mechanical Strength from Human iPSCs. *Cell Stem Cell*. 2020;26(2):251-261.e8. doi:10.1016/j.stem.2019.12.012
97. de Almeida PE, Ransohoff JD, Nahid A, Wu JC. Immunogenicity of Pluripotent Stem Cells and Their Derivatives. *Circ Res*. 2013;112(3):549-561. doi:10.1161/CIRCRESAHA.111.249243
98. Petrus-Reurer S, Romano M, Howlett S, Jones JL, Lombardi G, Saeb-Parsy K. Immunological considerations and challenges for regenerative cellular therapies. *Commun Biol*. 2021;4(1):798. doi:10.1038/s42003-021-02237-4
99. Jin X, Lin T, Xu Y. Stem Cell Therapy and Immunological Rejection in Animal Models. *Curr Mol Pharmacol*. 2016;9(4):284-288. doi:10.2174/1874467208666150928153511
100. Riolobos L, Hirata RK, Turtle CJ, et al. HLA Engineering of Human Pluripotent Stem Cells. *Mol Ther*. 2013;21(6):1232-1241. doi:10.1038/mt.2013.59
101. Li N, Rickel AP, Sanyour HJ, Hong Z. Vessel graft fabricated by the on-site differentiation of human mesenchymal stem cells towards vascular cells on vascular extracellular matrix scaffold under mechanical stimulation in a rotary bioreactor. *J Mater Chem B*. 2019;7(16):2703-2713. doi:10.1039/c8tb03348j
102. Xing Q, Qian Z, Tahtinen M, Yap AH, Yates K, Zhao F. Aligned Nanofibrous Cell-Derived Extracellular Matrix for Anisotropic Vascular Graft Construction. *Adv Healthc Mater*. 2017;6(10):10.1002/adhm.201601333. doi:10.1002/adhm.201601333

103. Kurobe H, Maxfield MW, Naito Y, et al. Comparison of a Closed System to a Standard Open Technique for Preparing Tissue-Engineered Vascular Grafts. *Tissue Eng Part C Methods*. 2015;21(1):88-93. doi:10.1089/ten.tec.2014.0160
104. Huang AH, Balestrini JL, Udelsman BV, et al. Biaxial Stretch Improves Elastic Fiber Maturation, Collagen Arrangement, and Mechanical Properties in Engineered Arteries. *Tissue Eng Part C Methods*. 2016;22(6):524-533. doi:10.1089/ten.TEC.2015.0309
105. Huang AH, Lee YU, Calle EA, et al. Design and Use of a Novel Bioreactor for Regeneration of Biaxially Stretched Tissue-Engineered Vessels. *Tissue Eng Part C Methods*. 2015;21(8):841-851. doi:10.1089/ten.TEC.2014.0287
106. Cunnane EM, Lorentz KL, Soletti L, et al. Development of a Semi-Automated, Bulk Seeding Device for Large Animal Model Implantation of Tissue Engineered Vascular Grafts. *Front Bioeng Biotechnol*. 2020;8:597847. doi:10.3389/fbioe.2020.597847
107. Håkansson J, Simsa R, Bogestål Y, et al. Individualized tissue-engineered veins as vascular grafts: A proof of concept study in pig. *J Tissue Eng Regen Med*. 2021;15(10):818-830. doi:10.1002/term.3233
108. Qiu X, Lee BLP, Wong SY, et al. Cellular remodeling of fibrotic conduit as vascular graft. *Biomaterials*. 2021;268:120565. doi:10.1016/j.biomaterials.2020.120565
109. Wang T, Dong N, Yan H, et al. Regeneration of a neoartery through a completely autologous acellular conduit in a minipig model: a pilot study. *J Transl Med*. 2019;17(1):24. doi:10.1186/s12967-018-1763-5
110. Fujita S, Yamagishi M, Kanda K, et al. Histology and Mechanics of In Vivo Tissue-Engineered Vascular Graft for Children. *Ann Thorac Surg*. 2020;110(3):1050-1054. doi:10.1016/j.athoracsur.2020.03.069
111. Nakayama Y, Kaneko Y, Okumura N, Terazawa T. Initial 3-year results of first human use of an in-body tissue-engineered autologous "Biotube" vascular graft for hemodialysis. *J Vasc Access*. 2020;21(1):110-115. doi:10.1177/1129729819852550
112. Chen CL, Guo HR, Wang YJ, et al. Combination of inductive effect of lipopolysaccharide and in situ mechanical conditioning for forming an autologous vascular graft in vivo. *Sci Rep*. 2019;9(1):10616. doi:10.1038/s41598-019-47054-2
113. Cunnane EM, Lorentz KL, Ramaswamy AK, et al. Extracellular Vesicles Enhance the Remodeling of Cell-Free Silk Vascular Scaffolds in Rat Aortae. *ACS Appl Mater Interfaces*. 2020;12(24):26955-26965. doi:10.1021/acsami.0c06609
114. Cunnane EM, Weinbaum JS, O'Brien FJ, Vorp DA. Future Perspectives on the Role of Stem Cells and Extracellular Vesicles in Vascular Tissue Regeneration. *Front Cardiovasc Med*. 2018;5:86. doi:10.3389/fcvm.2018.00086
115. Chen W, Yang M, Bai J, et al. Exosome-Modified Tissue Engineered Blood Vessel for Endothelial Progenitor Cell Capture and Targeted siRNA Delivery. *Macromol Biosci*. 2018;18(2):1700242. doi:10.1002/mabi.201700242
116. Ahangar P, Mills SJ, Cowin AJ. Mesenchymal Stem Cell Secretome as an Emerging Cell-Free Alternative for Improving Wound Repair. *Int J Mol Sci*. 2020;21(19):7038. doi:10.3390/ijms21197038
117. Baruah J, Wary KK. Exosomes in the Regulation of Vascular Endothelial Cell Regeneration. *Front Cell Dev Biol*. 2020;7:353. doi:10.3389/fcell.2019.00353
118. Nikfarjam S, Rezaie J, Zolbanin NM, Jafari R. Mesenchymal stem cell derived-exosomes: a modern approach in translational medicine. *J Transl Med*. 2020;18(1):449. doi:10.1186/s12967-020-02622-3

119. Tang Y, Zhou Y, Li HJ. Advances in mesenchymal stem cell exosomes: a review. *Stem Cell Res Ther*. 2021;12(1):71. doi:10.1186/s13287-021-02138-7
120. Hu S, Li Z, Shen D, et al. Exosome-eluting stents for vascular healing after ischaemic injury. *Nat Biomed Eng*. 2021;5(10):1174-1188. doi:10.1038/s41551-021-00705-0
121. Li X, Ma T, Sun J, et al. Harnessing the secretome of adipose-derived stem cells in the treatment of ischemic heart diseases. *Stem Cell Res Ther*. 2019;10(1):1-13. doi:10.1186/s13287-019-1289-7
122. Khosravi R, Ramachandra AB, Szafron JM, Schiavazzi DE, Breuer CK, Humphrey JD. A computational bio-chemo-mechanical model of in vivo tissue-engineered vascular graft development. *Integr Biol Quant Biosci Nano Macro*. 2020;12(3):47-63. doi:10.1093/intbio/zyaa004
123. Keshavarzian M, Meyer CA, Hayenga HN. In Silico Tissue Engineering: A Coupled Agent-Based Finite Element Approach. *Tissue Eng Part C Methods*. 2019;25(11):641-654. doi:10.1089/ten.TEC.2019.0103
124. Tamimi EA, Ardila DC, Ensley BD, Kellar RS, Vande Geest JP. Computationally Optimizing the Compliance of Multilayered Biomimetic Tissue Engineered Vascular Grafts. *J Biomech Eng*. 2019;141(6). doi:10.1115/1.4042902
125. Best C, Strouse R, Hor K, et al. Toward a patient-specific tissue engineered vascular graft. *J Tissue Eng*. 2018;9:2041731418764709. doi:10.1177/2041731418764709
126. Best CA, Szafron JM, Rocco KA, et al. Differential outcomes of venous and arterial tissue engineered vascular grafts highlight the importance of coupling long-term implantation studies with computational modeling. *Acta Biomater*. 2019;94:183-194. doi:10.1016/j.actbio.2019.05.063
127. Szafron JM, Ramachandra AB, Breuer CK, Marsden AL, Humphrey JD. Optimization of Tissue-Engineered Vascular Graft Design Using Computational Modeling. *Tissue Eng Part C Methods*. 2019;25(10):561-570. doi:10.1089/ten.tec.2019.0086
128. Furdella KJ, Higuchi S, Behrangzade A, Kim K, Wagner WR, Vande Geest JP. In-vivo assessment of a tissue engineered vascular graft computationally optimized for target vessel compliance. *Acta Biomater*. 2021;123:298-311. doi:10.1016/j.actbio.2020.12.058
129. Szafron JM, Khosravi R, Reinhardt J, et al. Immuno-driven and Mechano-mediated Neotissue Formation in Tissue Engineered Vascular Grafts. *Ann Biomed Eng*. 2018;46(11):1938-1950. doi:10.1007/s10439-018-2086-7
130. Zbinden JC, Blum KM, Berman AG, et al. Effects of Braiding Parameters on Tissue Engineered Vascular Graft Development. *Adv Healthc Mater*. 2020;9(24):2001093. doi:10.1002/adhm.202001093
131. Fukunishi T, Ong CS, Yesantharao P, et al. Different degradation rates of nanofiber vascular grafts in small and large animal models. *J Tissue Eng Regen Med*. 2020;14(2):203-214. doi:10.1002/term.2977
132. Stowell CET, Li X, Matsunaga MH, et al. Resorbable vascular grafts show rapid cellularization and degradation in the ovine carotid. *J Tissue Eng Regen Med*. 2020;14(11):1673-1684. doi:10.1002/term.3128
133. von Bornstädt D, Wang H, Paulsen MJ, et al. Rapid Self-Assembly of Bioengineered Cardiovascular Bypass Grafts From Scaffold-Stabilized, Tubular Bilevel Cell Sheets. *Circulation*. 2018;138(19):2130-2144. doi:10.1161/CIRCULATIONAHA.118.035231

134. Domínguez-Robles J, Shen T, Cornelius VA, et al. Development of drug loaded cardiovascular prosthesis for thrombosis prevention using 3D printing. *Mater Sci Eng C Mater Biol Appl*. 2021;129:112375. doi:10.1016/j.msec.2021.112375
135. Zhang X, Shi J, Chen S, et al. Polycaprolactone/gelatin degradable vascular grafts simulating endothelium functions modified by nitric oxide generation. *Regen Med*. 2019;14(12):1089-1105. doi:10.2217/rme-2019-0015
136. Kimicata M, Swamykumar P, Fisher JP. Extracellular Matrix for Small-Diameter Vascular Grafts. *Tissue Eng Part A*. 2020;26(23-24):1388-1401. doi:10.1089/ten.tea.2020.0201
137. Schneider KH, Enayati M, Grasl C, et al. Acellular vascular matrix grafts from human placenta chorion: Impact of ECM preservation on graft characteristics, protein composition and in vivo performance. *Biomaterials*. 2018;177:14-26. doi:10.1016/j.biomaterials.2018.05.045
138. Lopera Higuita M, Lopera Giraldo JF, Sarrafian TL, Griffiths LG. Tissue engineered bovine saphenous vein extracellular matrix scaffolds produced via antigen removal achieve high in vivo patency rates. *Acta Biomater*. 2021;134:144-159. doi:10.1016/j.actbio.2021.06.034
139. Valencia-Rivero KT, Cruz JC, Wagner-Gutierrez N, et al. Evaluation of Microscopic Structure–Function Relationships of PEGylated Small Intestinal Submucosa Vascular Grafts for Arteriovenous Connection. *ACS Appl Bio Mater*. 2019;2(9):3706-3721. doi:10.1021/acsabm.9b00158
140. Zhen L, Creason SA, Simonovsky FI, et al. Precision-porous polyurethane elastomers engineered for application in pro-healing vascular grafts: Synthesis, fabrication and detailed biocompatibility assessment. *Biomaterials*. 2021;279:121174. doi:10.1016/j.biomaterials.2021.121174
141. Dahan N, Sarig U, Bronshtein T, et al. Dynamic Autologous Reendothelialization of Small-Caliber Arterial Extracellular Matrix: A Preclinical Large Animal Study. *Tissue Eng Part A*. 2017;23(1-2):69-79. doi:10.1089/ten.TEA.2016.0126
142. Lin CH, Hsia K, Tsai CH, Ma H, Lu JH, Tsay RY. Decellularized porcine coronary artery with adipose stem cells for vascular tissue engineering. *Biomed Mater Bristol Engl*. 2019;14(4):045014. doi:10.1088/1748-605X/ab2329
143. Fayon A, Menu P, El Omar R. Cellularized small-caliber tissue-engineered vascular grafts: looking for the ultimate gold standard. *Npj Regen Med*. 2021;6(1):1-11. doi:10.1038/s41536-021-00155-x
144. Fukunishi T, Ong CS, He YJ, et al. Fast-Degrading Tissue-Engineered Vascular Grafts Lead to Increased Extracellular Matrix Cross-Linking Enzyme Expression. *Tissue Eng Part A*. 2021;27(21-22):1368-1375. doi:10.1089/ten.TEA.2020.0266
145. Fukunishi T, Ong CS, Lui C, et al. Formation of Neoarteries with Optimal Remodeling Using Rapidly Degrading Textile Vascular Grafts. *Tissue Eng Part A*. 2019;25(7-8):632-641. doi:10.1089/ten.TEA.2018.0167
146. Nguyen TU, Shojaee M, Bashur CA, Kishore V. Electrochemical fabrication of a biomimetic elastin-containing bi-layered scaffold for vascular tissue engineering. *Biofabrication*. 2018;11(1):015007. doi:10.1088/1758-5090/aaeab0
147. Akentjew TL, Terraza C, Suazo C, et al. Rapid fabrication of reinforced and cell-laden vascular grafts structurally inspired by human coronary arteries. *Nat Commun*. 2019;10(1):3098. doi:10.1038/s41467-019-11090-3
148. Fukunishi T, Best CA, Sugiura T, et al. Preclinical study of patient-specific cell-free nanofiber tissue-engineered vascular grafts using 3-dimensional printing in a sheep model. *J Thorac Cardiovasc Surg*. 2017;153(4):924-932. doi:10.1016/j.jtcvs.2016.10.066

149. Yang Y, Lei D, Zou H, et al. Hybrid electrospun rapamycin-loaded small-diameter decellularized vascular grafts effectively inhibit intimal hyperplasia. *Acta Biomater*. 2019;97:321-332. doi:10.1016/j.actbio.2019.06.037
150. Gong W, Lei D, Li S, et al. Hybrid small-diameter vascular grafts: Anti-expansion effect of electrospun poly ε-caprolactone on heparin-coated decellularized matrices. *Biomaterials*. 2016;76:359-370. doi:10.1016/j.biomaterials.2015.10.066
151. Ran X, Ye Z, Fu M, et al. Design, Preparation, and Performance of a Novel Bilayer Tissue-Engineered Small-Diameter Vascular Graft. *Macromol Biosci*. 2019;19(3):e1800189. doi:10.1002/mabi.201800189
152. Wu P, Nakamura N, Morita H, et al. A hybrid small-diameter tube fabricated from decellularized aortic intima-media and electrospun fiber for artificial small-diameter blood vessel. *J Biomed Mater Res A*. 2019;107(5):1064-1070. doi:10.1002/jbm.a.36631
153. Yi SW, Shin YM, Lee JB, et al. Dilation-Responsive Microshape Programing Prevents Vascular Graft Stenosis. *Small*. 2021;17(18):2007297. doi:10.1002/smll.202007297
154. Gupta P, Lorentz KL, Haskett DG, et al. Bioresorbable silk grafts for small diameter vascular tissue engineering applications: In vitro and in vivo functional analysis. *Acta Biomater*. 2020;105:146-158. doi:10.1016/j.actbio.2020.01.020
155. Tanaka T, Abe Y, Cheng CJ, Tanaka R, Naito A, Asakura T. Development of Small-Diameter Elastin-Silk Fibroin Vascular Grafts. *Front Bioeng Biotechnol*. 2020;8:622220. doi:10.3389/fbioe.2020.622220
156. Zamani M, Khafaji M, Naji M, Vossoughi M, Alemzadeh I, Haghighipour N. A Biomimetic Heparinized Composite Silk-Based Vascular Scaffold with sustained Antithrombogenicity. *Sci Rep*. 2017;7(1):4455. doi:10.1038/s41598-017-04510-1
157. Li X, Xu J, Nicolescu CT, Marinelli JT, Tien J. Generation, Endothelialization, and Microsurgical Suture Anastomosis of Strong 1-mm-Diameter Collagen Tubes. *Tissue Eng Part A*. 2017;23(7-8):335-344. doi:10.1089/ten.tea.2016.0339
158. Copes F, Pien N, Van Vlierberghe S, Boccafoschi F, Mantovani D. Collagen-Based Tissue Engineering Strategies for Vascular Medicine. *Front Bioeng Biotechnol*. 2019;7:166. doi:10.3389/fbioe.2019.00166
159. Syedain ZH, Graham ML, Dunn TB, et al. A completely biological "off-the-shelf" arteriovenous graft that recellularizes in baboons. *Sci Transl Med*. 2017;9(414):eaan4209. doi:10.1126/scitranslmed.aan4209
160. Mrówczyński W, Mugnai D, de Valence S, et al. Porcine carotid artery replacement with biodegradable electrospun poly-e-caprolactone vascular prosthesis. *J Vasc Surg*. 2014;59(1):210-219. doi:10.1016/j.jvs.2013.03.004
161. Sugiura T, Tara S, Nakayama H, et al. Fast-degrading bioresorbable arterial vascular graft with high cellular infiltration inhibits calcification of the graft. *J Vasc Surg*. 2017;66(1):243-250. doi:10.1016/j.jvs.2016.05.096
162. Fathi-Karkan S, Banimohamad-Shotorbani B, Saghati S, Rahbarghazi R, Davaran S. A critical review of fibrous polyurethane-based vascular tissue engineering scaffolds. *J Biol Eng*. 2022;16(1):6. doi:10.1186/s13036-022-00286-9
163. Eilenberg M, Enayati M, Ehebruster D, et al. Long Term Evaluation of Nanofibrous, Bioabsorbable Polycarbonate Urethane Grafts for Small Diameter Vessel Replacement in Rodents. *Eur J Vasc Endovasc Surg*. 2020;59(4):643-652. doi:10.1016/j.ejvs.2019.11.004
164. Zhu M, Wu Y, Li W, et al. Biodegradable and elastomeric vascular grafts enable vascular remodeling. *Biomaterials*. 2018;183:306-318. doi:10.1016/j.biomaterials.2018.08.063

165. Niklason LE, Lawson JH. Bioengineered human blood vessels. *Science*. 2020;370(6513):eaaw8682. doi:10.1126/science.aaw8682
166. Heng JW, Yazid MD, Abdul Rahman MR, Sulaiman N. Coatings in Decellularized Vascular Scaffolds for the Establishment of a Functional Endothelium: A Scoping Review of Vascular Graft Refinement. *Front Cardiovasc Med*. 2021;8:677588. doi:10.3389/fcvm.2021.677588
167. Syedain ZH, Maciver R, Tranquillo RT. Vascular grafts and valves that animate, made from decellularized biologically-engineered tissue tubes. *J Cardiovasc Surg (Torino)*. 2020;61(5):577-585. doi:10.23736/S0021-9509.20.11615-X
168. Gupta P, Mandal BB. Tissue-Engineered Vascular Grafts: Emerging Trends and Technologies. *Adv Funct Mater*. 2021;31(33):2100027. doi:10.1002/adfm.202100027
169. Gentile P, Sterodimas A, Pizzicannella J, et al. Systematic Review: Allogenic Use of Stromal Vascular Fraction (SVF) and Decellularized Extracellular Matrices (ECM) as Advanced Therapy Medicinal Products (ATMP) in Tissue Regeneration. *Int J Mol Sci*. 2020;21(14):E4982. doi:10.3390/ijms21144982
170. Yuan H, Chen C, Liu Y, Lu T, Wu Z. Strategies in cell-free tissue-engineered vascular grafts. *J Biomed Mater Res A*. 2020;108(3):426-445. doi:10.1002/jbm.a.36825
171. Zhang Q, Bosch-Rué È, Pérez RA, Truskey GA. Biofabrication of tissue engineering vascular systems. *APL Bioeng*. 2021;5(2):021507. doi:10.1063/5.0039628
172. Song HHG, Rumma RT, Ozaki CK, Edelman ER, Chen CS. Vascular Tissue Engineering: Progress, Challenges, and Clinical Promise. *Cell Stem Cell*. 2018;22(3):340-354. doi:10.1016/j.stem.2018.02.009
173. Hiob MA, She S, Muiznieks LD, Weiss AS. Biomaterials and Modifications in the Development of Small-Diameter Vascular Grafts. *ACS Biomater Sci Eng*. 2017;3(5):712-723. doi:10.1021/acsbiomaterials.6b00220
174. Pagel M, Beck-Sickinger AG. Multifunctional biomaterial coatings: synthetic challenges and biological activity. *Biol Chem*. 2017;398(1):3-22. doi:10.1515/hsz-2016-0204
175. Chandra P, Atala A. Engineering blood vessels and vascularized tissues: technology trends and potential clinical applications. *Clin Sci Lond Engl 1979*. 2019;133(9):1115-1135. doi:10.1042/CS20180155
176. Tseng CN, Karlöf E, Chang YT, et al. Contribution of Endothelial Injury and Inflammation in Early Phase to Vein Graft Failure: The Causal Factors Impact on the Development of Intimal Hyperplasia in Murine Models. *PLOS ONE*. 2014;9(6):e98904. doi:10.1371/journal.pone.0098904
177. Wissing TB, Bonito V, Bouten CVC, Smits AIPM. Biomaterial-driven in situ cardiovascular tissue engineering—a multi-disciplinary perspective. *NPJ Regen Med*. 2017;2:18. doi:10.1038/s41536-017-0023-2
178. Roh JD, Sawh-Martinez R, Brennan MP, et al. Tissue-engineered vascular grafts transform into mature blood vessels via an inflammation-mediated process of vascular remodeling. *Proc Natl Acad Sci U S A*. 2010;107(10):4669-4674. doi:10.1073/pnas.0911465107
179. Talacua H, Smits AIPM, Muylaert DEP, et al. In Situ Tissue Engineering of Functional Small-Diameter Blood Vessels by Host Circulating Cells Only. *Tissue Eng Part A*. 2015;21(19-20):2583-2594. doi:10.1089/ten.TEA.2015.0066
180. Julier Z, Park AJ, Briquez PS, Martino MM. Promoting tissue regeneration by modulating the immune system. *Acta Biomater*. 2017;53:13-28. doi:10.1016/j.actbio.2017.01.056

181. Boccafoschi F, Mosca C, Cannas M. Cardiovascular biomaterials: when the inflammatory response helps to efficiently restore tissue functionality? *J Tissue Eng Regen Med*. 2014;8(4):253-267. doi:10.1002/term.1526
182. Rodriguez-Soto MA, Suarez Vargas N, Riveros A, et al. Failure Analysis of TEVG's I: Overcoming the Initial Stages of Blood Material Interaction and Stabilization of the Immune Response. *Cells*. 2021;10(11):3140. doi:10.3390/cells10113140
183. Boccafoschi F, Mosca C, Cannas M. Cardiovascular biomaterials: when the inflammatory response helps to efficiently restore tissue functionality? *J Tissue Eng Regen Med*. 2014;8(4):253-267. doi:10.1002/term.1526
184. Browne S, Pandit A. Biomaterial-Mediated Modification of the Local Inflammatory Environment. *Front Bioeng Biotechnol*. 2015;3:67. doi:10.3389/fbioe.2015.00067
185. Zhang F, King MW. Immunomodulation Strategies for the Successful Regeneration of a Tissue-Engineered Vascular Graft. *Adv Healthc Mater*. 2022;11(12):2200045. doi:10.1002/adhm.202200045
186. Chang YC, Li J, Mirhaidari G, et al. Zoledronate alters natural progression of tissue-engineered vascular grafts. *FASEB J Off Publ Fed Am Soc Exp Biol*. 2021;35(10):e21849. doi:10.1096/fj.202001606RR
187. Ruiz-Rosado J de D, Lee YU, Mahler N, et al. Angiotensin II receptor I blockade prevents stenosis of tissue engineered vascular grafts. *FASEB J Off Publ Fed Am Soc Exp Biol*. Published online June 15, 2018:fj201800458. doi:10.1096/fj.201800458
188. Shi J, Zhang X, Jiang L, et al. Regulation of the inflammatory response by vascular grafts modified with Aspirin-Triggered Resolvin D1 promotes blood vessel regeneration. *Acta Biomater*. 2019;97:360-373. doi:10.1016/j.actbio.2019.07.037
189. Tan RP, Chan AHP, Wei S, et al. Bioactive Materials Facilitating Targeted Local Modulation of Inflammation. *JACC Basic Transl Sci*. 2019;4(1):56-71. doi:10.1016/j.jacbts.2018.10.004
190. Gupta P, Chaudhuri GR, Janani G, et al. Functionalized Silk Vascular Grafts with Decellularized Human Wharton's Jelly Improves Remodeling via Immunomodulation in Rabbit Jugular Vein. *Adv Healthc Mater*. 2021;10(19):e2100750. doi:10.1002/adhm.202100750
191. Lorentz KL, Gupta P, Shehabeldin MS, et al. CCL2 loaded microparticles promote acute patency in silk-based vascular grafts implanted in rat aortae. *Acta Biomater*. 2021;135:126-138. doi:10.1016/j.actbio.2021.08.049
192. Shafiq M, Zhang Q, Zhi D, et al. In Situ Blood Vessel Regeneration Using SP (Substance P) and SDF (Stromal Cell–Derived Factor)-1α Peptide Eluting Vascular Grafts. *Arterioscler Thromb Vasc Biol*. 2018;38(7):e117-e134. doi:10.1161/ATVBAHA.118.310934
193. Kim D, Chung JJ, Jung Y, Kim SH. The effect of Substance P/Heparin conjugated PLCL polymer coating of bioinert ePTFE vascular grafts on the recruitment of both ECs and SMCs for accelerated regeneration. *Sci Rep*. 2019;9(1):17083. doi:10.1038/s41598-019-53514-6
194. Fang S, Ahlmann AH, Langhorn L, et al. Small diameter polycaprolactone vascular grafts are patent in sheep carotid bypass but require antithrombotic therapy. *Regen Med*. Published online March 25, 2021. doi:10.2217/rme-2020-0171
195. Radke D, Jia W, Sharma D, et al. Tissue Engineering at the Blood-Contacting Surface: A Review of Challenges and Strategies in Vascular Graft Development. *Adv Healthc Mater*. 2018;7(15):e1701461. doi:10.1002/adhm.201701461

196. Strang AC, Knetsch MLW, Idu MM, et al. Superior in vivo compatibility of hydrophilic polymer coated prosthetic vascular grafts. *J Vasc Access*. 2014;15(2):95-101. doi:10.5301/jva.5000166
197. Thalla PK, Contreras-García A, Fadlallah H, et al. A Versatile Star PEG Grafting Method for the Generation of Nonfouling and Nonthrombogenic Surfaces. *BioMed Res Int*. 2012;2013:e962376. doi:10.1155/2013/962376
198. Iglesias-Echevarria M, Johnson R, Rafuse M, Ding Y, Tan W. Vascular Grafts with Tailored Stiffness and a Ligand Environment via Multiarmed Polymer Sheath for Expeditious Regeneration. *ACS Appl Bio Mater*. 2021;4(1):545-558. doi:10.1021/acsabm.0c01114
199. Poterucha TJ, Libby P, Goldhaber SZ. More than an anticoagulant: Do heparins have direct anti-inflammatory effects? *Thromb Haemost*. 2017;117(3):437-444. doi:10.1160/TH16-08-0620
200. Jiang B, Suen R, Wang JJ, Zhang ZJ, Wertheim JA, Ameer GA. Mechanocompatible Polymer-Extracellular Matrix Composites for Vascular Tissue Engineering. *Adv Healthc Mater*. 2016;5(13):1594-1605. doi:10.1002/adhm.201501003
201. Freeman J, Chen A, Weinberg RJ, Okada T, Chen C, Lin PH. Sustained Thromboresistant Bioactivity with Reduced Intimal Hyperplasia of Heparin-Bonded Polytetrafluoroethylene Propaten Graft in a Chronic Canine Femoral Artery Bypass Model. *Ann Vasc Surg*. 2018;49:295-303. doi:10.1016/j.avsg.2017.09.017
202. Samson RH, Morales R, Showalter DP, Lepore MR, Nair DG. Heparin-bonded expanded polytetrafluoroethylene femoropopliteal bypass grafts outperform expanded polytetrafluoroethylene grafts without heparin in a long-term comparison. *J Vasc Surg*. 2016;64(3):638-647. doi:10.1016/j.jvs.2016.03.414
203. Matsuzaki Y, Miyamoto S, Miyachi H, et al. Improvement of a Novel Small-diameter Tissue-engineered Arterial Graft With Heparin Conjugation. *Ann Thorac Surg*. 2021;111(4):1234-1241. doi:10.1016/j.athoracsur.2020.06.112
204. Qiu X, Lee BLP, Ning X, Murthy N, Dong N, Li S. End-point immobilization of heparin on plasma-treated surface of electrospun polycarbonate-urethane vascular graft. *Acta Biomater*. 2017;51:138-147. doi:10.1016/j.actbio.2017.01.012
205. Shi J, Chen S, Wang L, et al. Rapid endothelialization and controlled smooth muscle regeneration by electrospun heparin-loaded polycaprolactone/gelatin hybrid vascular grafts. *J Biomed Mater Res B Appl Biomater*. 2019;107(6):2040-2049. doi:10.1002/jbm.b.34295
206. Xu Z, Gu Y, Li J, et al. Vascular Remodeling Process of Heparin-Conjugated Poly(ε-Caprolactone) Scaffold in a Rat Abdominal Aorta Replacement Model. *J Vasc Res*. 2018;55(6):338-349. doi:10.1159/000494509
207. Zhu T, Gu H, Zhang H, et al. Covalent grafting of PEG and heparin improves biological performance of electrospun vascular grafts for carotid artery replacement. *Acta Biomater*. 2021;119:211-224. doi:10.1016/j.actbio.2020.11.013
208. Kong X, Kong C, Wen S, Shi J. The use of heparin, bFGF, and VEGF 145 grafted acellular vascular scaffold in small diameter vascular graft. *J Biomed Mater Res B Appl Biomater*. 2019;107(3):672-679. doi:10.1002/jbm.b.34160
209. Lee KS, Kayumov M, Emechebe GA, et al. A Comparative Study of an Anti-Thrombotic Small-Diameter Vascular Graft with Commercially Available e-PTFE Graft in a Porcine Carotid Model. *Tissue Eng Regen Med*. 2022;19(3):537-551. doi:10.1007/s13770-021-00422-4

210. Dimitrievska S, Wang J, Lin T, et al. Glycocalyx-Like Hydrogel Coatings for Small Diameter Vascular Grafts. *Adv Funct Mater*. 2020;30(23):1908963. doi:10.1002/adfm.201908963
211. Kristofik NJ, Qin L, Calabro NE, et al. Improving in vivo outcomes of decellularized vascular grafts via incorporation of a novel extracellular matrix. *Biomaterials*. 2017;141:63-73. doi:10.1016/j.biomaterials.2017.06.025
212. Heath DE. Promoting Endothelialization of Polymeric Cardiovascular Biomaterials. *Macromol Chem Phys*. 2017;218(8):1600574. doi:10.1002/macp.201600574
213. Zilla P, Bezuidenhout D, Human P. Prosthetic vascular grafts: wrong models, wrong questions and no healing. *Biomaterials*. 2007;28(34):5009-5027. doi:10.1016/j.biomaterials.2007.07.017
214. Pennel T, Zilla P, Bezuidenhout D. Differentiating transmural from transanastomotic prosthetic graft endothelialization through an isolation loop-graft model. *J Vasc Surg*. 2013;58(4):1053-1061. doi:10.1016/j.jvs.2012.11.093
215. Lu S, Zhang P, Sun X, et al. Synthetic ePTFE grafts coated with an anti-CD133 antibody-functionalized heparin/collagen multilayer with rapid in vivo endothelialization properties. *ACS Appl Mater Interfaces*. 2013;5(15):7360-7369. doi:10.1021/am401706w
216. Hao D, Fan Y, Xiao W, et al. Rapid endothelialization of small diameter vascular grafts by a bioactive integrin-binding ligand specifically targeting endothelial progenitor cells and endothelial cells. *Acta Biomater*. 2020;108:178-193. doi:10.1016/j.actbio.2020.03.005
217. Choi WS, Joung YK, Lee Y, et al. Enhanced Patency and Endothelialization of Small-Caliber Vascular Grafts Fabricated by Coimmobilization of Heparin and Cell-Adhesive Peptides. *ACS Appl Mater Interfaces*. 2016;8(7):4336-4346. doi:10.1021/acsami.5b12052
218. Hao D, Xiao W, Liu R, et al. Discovery and Characterization of a Potent and Specific Peptide Ligand Targeting Endothelial Progenitor Cells and Endothelial Cells for Tissue Regeneration. *ACS Chem Biol*. 2017;12(4):1075-1086. doi:10.1021/acschembio.7b00118
219. Liu Y, Mahara A, Kambe Y, Hsu YI, Yamaoka T. Endothelial cell adhesion and blood response to hemocompatible peptide 1 (HCP-1), REDV, and RGD peptide sequences with free N-terminal amino groups immobilized on a biomedical expanded polytetrafluorethylene surface. *Biomater Sci*. 2021;9(3):1034-1043. doi:10.1039/d0bm01396j
220. Olausson M, Patil PB, Kuna VK, et al. Transplantation of an allogeneic vein bioengineered with autologous stem cells: a proof-of-concept study. *Lancet Lond Engl*. 2012;380(9838):230-237. doi:10.1016/S0140-6736(12)60633-3
221. Ardila DC, Liou JJ, Maestas D, et al. Surface Modification of Electrospun Scaffolds for Endothelialization of Tissue-Engineered Vascular Grafts Using Human Cord Blood-Derived Endothelial Cells. *J Clin Med*. 2019;8(2):E185. doi:10.3390/jcm8020185
222. Sánchez PF, Brey EM, Briceño JC. Endothelialization mechanisms in vascular grafts. *J Tissue Eng Regen Med*. 2018;12(11):2164-2178. doi:10.1002/term.2747
223. Wang D, Wang X, Zhang Z, et al. Programmed Release of Multimodal, Cross-Linked Vascular Endothelial Growth Factor and Heparin Layers on Electrospun Polycaprolactone Vascular Grafts. *ACS Appl Mater Interfaces*. 2019;11(35):32533-32542. doi:10.1021/acsami.9b10621
224. Koobatian MT, Row S, Smith RJ, Koenigsknecht C, Andreadis ST, Swartz DD. Successful endothelialization and remodeling of a cell-free small-diameter arterial graft in a large animal model. *Biomaterials*. 2016;76:344-358. doi:10.1016/j.biomaterials.2015.10.020

225. Heidenhain C, Veeravoorn A, Vachkov B, et al. Fibroblast and vascular endothelial growth factor coating of decellularized vascular grafts stimulates undesired giant cells and graft encapsulation in a rat model. *Artif Organs*. 2011;35(1):E1-10. doi:10.1111/j.1525-1594.2010.01072.x
226. Antonova L, Kutikhin A, Sevostianova V, et al. bFGF and SDF-1α Improve In Vivo Performance of VEGF-Incorporating Small-Diameter Vascular Grafts. *Pharm Basel Switz*. 2021;14(4):302. doi:10.3390/ph14040302
227. Kong X, Kong C, Wen S, Shi J. The use of heparin, bFGF, and VEGF 145 grafted acellular vascular scaffold in small diameter vascular graft. *J Biomed Mater Res B Appl Biomater*. 2019;107(3):672-679. doi:10.1002/jbm.b.34160
228. Smith RJ, Nasiri B, Kann J, et al. Endothelialization of arterial vascular grafts by circulating monocytes. *Nat Commun*. 2020;11(1):1622. doi:10.1038/s41467-020-15361-2
229. Smith Jr. RJ, Yi T, Nasiri B, Breuer CK, Andreadis ST. Implantation of VEGF-functionalized cell-free vascular grafts: regenerative and immunological response. *FASEB J*. 2019;33(4):5089-5100. doi:10.1096/fj.201801856R
230. Zeng W, Wen C, Wu Y, et al. The use of BDNF to enhance the patency rate of small-diameter tissue-engineered blood vessels through stem cell homing mechanisms. *Biomaterials*. 2012;33(2):473-484. doi:10.1016/j.biomaterials.2011.09.066
231. Issa Bhaloo S, Wu Y, Le Bras A, et al. Binding of Dickkopf-3 to CXCR7 Enhances Vascular Progenitor Cell Migration and Degradable Graft Regeneration. *Circ Res*. 2018;123(4):451-466. doi:10.1161/CIRCRESAHA.118.312945
232. Wang W, Liu D, Li D, et al. Nanofibrous vascular scaffold prepared from miscible polymer blend with heparin/stromal cell-derived factor-1 alpha for enhancing anticoagulation and endothelialization. *Colloids Surf B Biointerfaces*. 2019;181:963-972. doi:10.1016/j.colsurfb.2019.06.065
233. Gao A, Hang R, Li W, et al. Linker-free covalent immobilization of heparin, SDF-1α, and CD47 on PTFE surface for antithrombogenicity, endothelialization and anti-inflammation. *Biomaterials*. 2017;140:201-211. doi:10.1016/j.biomaterials.2017.06.023
234. Hytönen JP, Leppänen O, Taavitsainen J, et al. Improved endothelialization of small-diameter ePTFE vascular grafts through growth factor therapy. *Vasc Biol*. 2019;1(1):1-9. doi:10.1530/VB-18-0001
235. Zhou F, Jia X, Yang Y, et al. Nanofiber-mediated microRNA-126 delivery to vascular endothelial cells for blood vessel regeneration. *Acta Biomater*. 2016;43:303-313. doi:10.1016/j.actbio.2016.07.048
236. Wen M, Zhi D, Wang L, et al. Local Delivery of Dual MicroRNAs in Trilayered Electrospun Grafts for Vascular Regeneration. *ACS Appl Mater Interfaces*. 2020;12(6):6863-6875. doi:10.1021/acsami.9b19452
237. Washington KS, Bashur CA. Delivery of Antioxidant and Anti-inflammatory Agents for Tissue Engineered Vascular Grafts. *Front Pharmacol*. 2017;8:659. doi:10.3389/fphar.2017.00659
238. Strijdom H, Chamane N, Lochner A. Nitric oxide in the cardiovascular system: a simple molecule with complex actions. *Cardiovasc J Afr*. 2009;20(5):303-310.
239. Napoli C, de Nigris F, Williams-Ignarro S, Pignalosa O, Sica V, Ignarro LJ. Nitric oxide and atherosclerosis: an update. *Nitric Oxide Biol Chem*. 2006;15(4):265-279. doi:10.1016/j.niox.2006.03.011

240. Devine R, Goudie MJ, Singha P, et al. Mimicking the Endothelium: Dual Action Heparinized Nitric Oxide Releasing Surface. *ACS Appl Mater Interfaces*. 2020;12(18):20158-20171. doi:10.1021/acsami.9b22277
241. Dou J, Wang Y, Jin X, et al. PCL/sulfonated keratin mats for vascular tissue engineering scaffold with potential of catalytic nitric oxide generation. *Mater Sci Eng C Mater Biol Appl*. 2020;107:110246. doi:10.1016/j.msec.2019.110246
242. Li P, Wang Y, Jin X, et al. Catalytic Generation of Nitric Oxide from Poly(ε-caprolactone)/Phosphobetainized Keratin Mats for a Vascular Tissue Engineering Scaffold. *Langmuir ACS J Surf Colloids*. 2020;36(16):4396-4404. doi:10.1021/acs.langmuir.0c00579
243. Enayati M, Schneider KH, Almeria C, et al. S-nitroso human serum albumin as a nitric oxide donor in drug-eluting vascular grafts: Biofunctionality and preclinical evaluation. *Acta Biomater*. 2021;134:276-288. doi:10.1016/j.actbio.2021.07.048
244. Tang D, Chen S, Hou D, et al. Regulation of macrophage polarization and promotion of endothelialization by NO generating and PEG-YIGSR modified vascular graft. *Mater Sci Eng C Mater Biol Appl*. 2018;84:1-11. doi:10.1016/j.msec.2017.11.005
245. Wang Y, Chen S, Pan Y, et al. Rapid in situ endothelialization of a small diameter vascular graft with catalytic nitric oxide generation and promoted endothelial cell adhesion. *J Mater Chem B*. 2015;3(47):9212-9222. doi:10.1039/C5TB02080H
246. Yang Z, Yang Y, Xiong K, et al. Nitric oxide producing coating mimicking endothelium function for multifunctional vascular stents. *Biomaterials*. 2015;63:80-92. doi:10.1016/j.biomaterials.2015.06.016
247. Yang Y, Gao P, Wang J, et al. Endothelium-Mimicking Multifunctional Coating Modified Cardiovascular Stents via a Stepwise Metal-Catechol-(Amine) Surface Engineering Strategy. *Research*. 2020;2020:9203906. doi:10.34133/2020/9203906
248. Jiang B, Suen R, Wang JJ, Zhang ZJ, Wertheim JA, Ameer GA. Vascular Scaffolds with Enhanced Antioxidant Activity Inhibit Graft Calcification. *Biomaterials*. 2017;144:166-175. doi:10.1016/j.biomaterials.2017.08.014
249. Strobel HA, Hookway TA, Piola M, et al. Assembly of Tissue-Engineered Blood Vessels with Spatially Controlled Heterogeneities. *Tissue Eng Part A*. 2018;24(19-20):1492-1503. doi:10.1089/ten.tea.2017.0492
250. ANSI/AAMI/ISO 7198:1998/2001 (R2010) - Cardiovascular implants - Tubular vascular prostheses. Accessed December 15, 2021. https://webstore.ansi.org/standards/aami/ansiaamiiso719819982001r2010
251. Guidelines | Vascular Access Society. Accessed December 13, 2021. https://www.vascularaccesssociety.com/education/guidelines
252. Wu DJ, van Dongen K, Szymczyk W, et al. Optimization of Anti-kinking Designs for Vascular Grafts Based on Supramolecular Materials. *Front Mater*. 2020;7:220. doi:10.3389/fmats.2020.00220
253. Itoh M, Mukae Y, Kitsuka T, et al. Development of an immunodeficient pig model allowing long-term accommodation of artificial human vascular tubes. *Nat Commun*. 2019;10(1):2244. doi:10.1038/s41467-019-10107-1
254. Cai Q, Liao W, Xue F, et al. Selection of different endothelialization modes and different seed cells for tissue-engineered vascular graft. *Bioact Mater*. 2021;6(8):2557-2568. doi:10.1016/j.bioactmat.2020.12.021

255. Syedain ZH, Graham ML, Dunn TB, et al. A completely biological "off-the-shelf" arteriovenous graft that recellularizes in baboons. *Sci Transl Med*. 2017;9(414):eaan4209. doi:10.1126/scitranslmed.aan4209
256. Rothuizen TC, Damanik FFR, Lavrijsen T, et al. Development and evaluation of in vivo tissue engineered blood vessels in a porcine model. *Biomaterials*. 2016;75:82-90. doi:10.1016/j.biomaterials.2015.10.023
257. Liu RH, Ong CS, Fukunishi T, Ong K, Hibino N. Review of Vascular Graft Studies in Large Animal Models. *Tissue Eng Part B Rev*. 2018;24(2):133-143. doi:10.1089/ten.teb.2017.0350
258. Schleimer K, Jalaie H, Afify M, et al. Sheep models for evaluation of novel patch and prosthesis material in vascular surgery: tips and tricks to avoid possible pitfalls. *Acta Vet Scand*. 2018;60:42. doi:10.1186/s13028-018-0397-1
259. Ma X, He Z, Li L, et al. Development and in vivo validation of tissue-engineered, small-diameter vascular grafts from decellularized aortae of fetal pigs and canine vascular endothelial cells. *J Cardiothorac Surg*. 2017;12(1):101. doi:10.1186/s13019-017-0661-x
260. Fukunishi T, Best CA, Sugiura T, et al. Tissue-Engineered Small Diameter Arterial Vascular Grafts from Cell-Free Nanofiber PCL/Chitosan Scaffolds in a Sheep Model. *PLOS ONE*. 2016;11(7):e0158555. doi:10.1371/journal.pone.0158555
261. Ju YM, Ahn H, Arenas-Herrera J, et al. Electrospun vascular scaffold for cellularized small diameter blood vessels: A preclinical large animal study. *Acta Biomater*. 2017;59:58-67. doi:10.1016/j.actbio.2017.06.027
262. Gage SM, Lawson JH. Bioengineered hemodialysis access grafts. *J Vasc Access*. 2017;18(Suppl. 1):56-63. doi:10.5301/jva.5000692
263. Ong CS, Fukunishi T, Liu RH, et al. Bilateral Arteriovenous Shunts as a Method for Evaluating Tissue-Engineered Vascular Grafts in Large Animal Models. *Tissue Eng Part C Methods*. 2017;23(11):728-735. doi:10.1089/ten.TEC.2017.0217
264. Maxfield MW, Stacy MR, Kurobe H, et al. Novel application and serial evaluation of tissue-engineered portal vein grafts in a murine model. *Regen Med*. 2017;12(8):929-938. doi:10.2217/rme-2017-0021
265. Hutchin P, Jacobs JR, Devin JB, Shaughnessy S, Roland AS. Bovine graft arteriovenous fistulas for maintenance hemodialysis. *Surg Gynecol Obstet*. 1975;141(2):255-258.
266. Arhuidese I, Reifsnyder T, Islam T, et al. Bovine carotid artery biologic graft outperforms expanded polytetrafluoroethylene for hemodialysis access. *J Vasc Surg*. 2017;65(3):775-782. doi:10.1016/j.jvs.2016.10.080
267. Kennealey PT, Elias N, Hertl M, et al. A prospective, randomized comparison of bovine carotid artery and expanded polytetrafluoroethylene for permanent hemodialysis vascular access. *J Vasc Surg*. 2011;53(6):1640-1648. doi:10.1016/j.jvs.2011.02.008
268. Lindsey P, Echeverria A, Cheung M, Kfoury E, Bechara CF, Lin PH. Lower Extremity Bypass Using Bovine Carotid Artery Graft (Artegraft): An Analysis of 124 Cases with Long-Term Results. *World J Surg*. 2018;42(1):295-301. doi:10.1007/s00268-017-4161-x
269. Dyer-Kindy LM, Heelan Gladden AA, Gralla J, et al. Relationship between bovine carotid artery grafts for hemodialysis access and human leukocyte antigen sensitization. *Hemodial Int Int Symp Home Hemodial*. 2020;24(1):36-42. doi:10.1111/hdi.12784
270. Hatzibaloglou A, Velissaris I, Kaitzis D, Grekas D, Avdelidou A, Kiskinis D. ProCol® Vascular Bioprosthesis for Vascular Access: Midterm Results. *J Vasc Access*. 2004;5(1):16-18. doi:10.1177/112972980400500104

271. Schmidli J, Savolainen H, Heller G, et al. Bovine mesenteric vein graft (ProCol) in critical limb ischaemia with tissue loss and infection. *Eur J Vasc Endovasc Surg Off J Eur Soc Vasc Surg*. 2004;27(3):251-253. doi:10.1016/j.ejvs.2003.12.001
272. Madden RL, Lipkowitz GS, Browne BJ, Kurbanov A. Experience with cryopreserved cadaveric femoral vein allografts used for hemodialysis access. *Ann Vasc Surg*. 2004;18(4):453-458. doi:10.1007/s10016-004-0055-0
273. Herrmann FEM, Lamm P, Wellmann P, Milz S, Hagl C, Juchem G. Autologous endothelialized vein allografts in coronary artery bypass surgery – Long term results. *Biomaterials*. 2019;212:87-97. doi:10.1016/j.biomaterials.2019.05.019
274. Merola J, Jane-Wit DD, Pober JS. Recent advances in allograft vasculopathy. *Curr Opin Organ Transplant*. 2017;22(1):1-7. doi:10.1097/MOT.0000000000000370
275. Jakimowicz T, Przywara S, Turek J, et al. Five Year Outcomes in Patients with End Stage Renal Disease Who Received a Bioengineered Human Acellular Vessel for Dialysis Access. *EJVES Vasc Forum*. 2022;54:58-63. doi:10.1016/j.ejvsvf.2022.01.003
276. Gutowski P, Gage SM, Guziewicz M, et al. Arterial reconstruction with human bioengineered acellular blood vessels in patients with peripheral arterial disease. *J Vasc Surg*. 2020;72(4):1247-1258. doi:10.1016/j.jvs.2019.11.056
277. Wystrychowski W, McAllister TN, Zagalski K, Dusserre N, Cierpka L, L'Heureux N. First human use of an allogeneic tissue-engineered vascular graft for hemodialysis access. *J Vasc Surg*. 2014;60(5):1353-1357. doi:10.1016/j.jvs.2013.08.018
278. Kato N, Yamagishi M, Kanda K, et al. First Successful Clinical Application of the In Vivo Tissue-Engineered Autologous Vascular Graft. *Ann Thorac Surg*. 2016;102(4):1387-1390. doi:10.1016/j.athoracsur.2016.06.095
279. Nakatsuji H, Yamagishi M, Maeda Y, et al. Midterm results of pulmonary artery plasty with in vivo tissue-engineered vascular grafts. *Interact Cardiovasc Thorac Surg*. 2021;32(6):956-959. doi:10.1093/icvts/ivab019
280. Higashita R, Miyazaki M, Oi M, Ishikawa N. The first-in-human results of an in-body tissue architecture-induced tissue engineered vascular graft "Biotube" for application in distal bypass for chronic limb-threatening ischemia. *J Vasc Surg Cases Innov Tech*. Published online July 31, 2022. doi:10.1016/j.jvscit.2022.07.007
281. Owens CD, Gasper WJ, Rahman AS, Conte MS. Vein graft failure. *J Vasc Surg*. 2015;61(1):203-216. doi:10.1016/j.jvs.2013.08.019
282. Rosa S, Praça C, Pitrez PR, et al. Functional characterization of iPSC-derived arterial- and venous-like endothelial cells. *Sci Rep*. 2019;9(1):3826. doi:10.1038/s41598-019-40417-9
